# Participation of the Transcriptional Regulator Fur in Modulating Biofilm Formation of the Salmonid Pathogen *Yersinia ruckeri*: Beyond Iron Homeostasis

**DOI:** 10.3390/ijms27188413

**Published:** 2026-09-21

**Authors:** Diego Peñaloza, María J. Barros, Fausto Cabezas-Mera, Lillian G. Acuña, Pía Vásquez-Arriagada, Francisco Parra, Camila Queraltó, Camila Silva, Simón Díaz, Claudia Ubilla, Ana Moya-Beltrán, Andrés Silva, Jan Nevermann, Pilar Parada, Fernando Gil, Juan A. Fuentes, Iván L. Calderón

**Affiliations:** 1 Laboratorio de RNAs Bacterianos, Centro de Investigación de Resiliencia a Pandemias, Facultad de Ciencias de la Vida, Universidad Andrés Bello, Santiago 8370186, Chile; diego.ignacio.mp@gmail.com (D.P.); mbarrosgamonal@gmail.com (M.J.B.); lillian.acuna@unab.cl (L.G.A.); piacatalina.vasquez@gmail.com (P.V.-A.); camilaabeleen25@gmail.com (C.Q.); c.silvacaldern@uandresbello.edu (C.S.); simon.diaz.palma@gmail.com (S.D.); c.ubillagonzlez@uandresbello.edu (C.U.); 2 Programa de Doctorado en Biotecnología, Facultad de Ciencias de la Vida, Universidad Andrés Bello, Santiago 8370186, Chile; f.parralathrop@gmail.com; 3 Programa de Doctorado en Informática Aplicada a Salud y Medio Ambiente, Escuela de Postgrado, Universidad Tecnológica Metropolitana, Santiago 8330300, Chile; fcabezasme@utem.cl; 4 Departamento de Ciencias Biológicas, Facultad de Ciencias de la Vida, Universidad Andrés Bello, Santiago 8370186, Chile; 5 Programa de Doctorado en Biociencias Moleculares, Facultad de Ciencias de la Vida, Universidad Andrés Bello, Santiago 8370186, Chile; 6 Laboratorio de Genética y Patogénesis Bacteriana, Centro de Investigación de Resiliencia a Pandemias, Facultad de Ciencias de la Vida, Universidad Andrés Bello, Santiago 8370186, Chile; a.silvapeailillo@uandresbello.edu (A.S.); j.nevermann@uandresbello.edu (J.N.); 7 Departamento de Informática y Computación, Facultad de Ingeniería, Universidad Tecnológica Metropolitana, Santiago 7800002, Chile; amoya@utem.cl; 8 Centre for Systems Biotechnology, Facultad de Ciencias de la Vida, Universidad Andrés Bello, Santiago 7550196, Chile; pilar.parada@unab.cl; 9 Microbiota-Host Interactions & Clostridia Research Group, Program in Microbiota and Applied Molecular Biology, Center for Biomedical Research and Innovation (CIIB), School of Medicine, Faculty of Medicine, Universidad de los Andes, Santiago 7620001, Chile; frgil@uandes.cl

**Keywords:** Fur, biofilm-cycle regulation, transcriptomic profiling, *Yersinia ruckeri*, salmonid pathogen

## Abstract

Biofilm formation by bacterial pathogens represents a major challenge in aquaculture, since biofilms promote persistence and increase tolerance to antimicrobial treatments. *Yersinia ruckeri*, the etiological agent of enteric redmouth disease in salmonids, contributes to recurrent infections in aquaculture. However, the molecular mechanisms governing biofilm development in this pathogen remain poorly understood. In this study, we investigated the role of the global transcriptional regulator Fur in the biofilm formation of *Y. ruckeri*. To this aim, a *fur* deletion mutant (Δ*fur*) was phenotypically, structurally, and transcriptionally characterized under biofilm-forming conditions. Fur deletion resulted in impaired motility, reduced flagellar synthesis, and a marked decrease in mature biofilm formation. These changes were accompanied by alterations in three-dimensional architecture, cellular organization, and extracellular matrix production, as revealed by scanning electron and confocal microscopy analyses. Furthermore, transcriptomic profiling via RNA-seq demonstrated that the absence of Fur leads to extensive transcriptional reprogramming. Genes associated with the biosynthesis of flagella and oxidative stress response were significantly down-regulated, whereas genes associated with SOS responses, non-ribosomal peptide biosynthesis, and those related to iron acquisition and siderophore systems exhibited significant up-regulation in the absence of Fur. Interestingly, the strong up-regulation of P2-type prophage genes was also observed in the Δ*fur* strain. Collectively, our findings identify Fur as an important contributor to the normal progression toward mature and spatially organized *Y. ruckeri* biofilms. Likewise, the Fur-dependent pathways and factors identified here provide candidates for future mechanistic studies and may ultimately help identify strategies to interfere with *Y. ruckeri* persistence in aquaculture environments.

## 1. Introduction

Biofilm development on abiotic surfaces, prevalent in clinical, industrial, and natural settings, facilitates pathogen tolerance to antimicrobial treatments and the acquisition of antibiotic resistance determinants [1,2]. In aquaculture environments, this phenomenon critically promotes pathogen persistence, thereby triggering recurrent infectious outbreaks [3]. The subsequent excessive use of antibiotics to prevent and control these infections inadvertently compromises fish production, disrupts ecological balances, and poses significant risks to human health [2,4]. *Yersinia ruckeri*, the etiological agent of enteric redmouth disease (ERM) in salmonids, forms biofilms on diverse abiotic surfaces commonly present in aquaculture facilities associated with different stages of fish growth, such as plastic polymers, fiberglass, and wood [5,6,7]. Consequently, this pathogen affects various developmental stages of salmonids, compromising fish health and production, as well as ecosystem integrity.

Bacterial biofilms are complex, three-dimensional communities embedded in a self-produced extracellular matrix of polymeric substances that are mainly made of polysaccharides, proteins, and extracellular DNA [8]. Biofilm formation is a cyclic process that occurs in a stage-specific and progressive manner [9]. This process initiates with the transition from a planktonic to a sessile lifestyle, characterized by reversible, followed by irreversible, adhesion of bacteria to the surface. This initial attachment depends on surface appendages, such as flagella and adhesins, which mediate motility and facilitate early interactions with the substrate [10]. Following adhesion, bacteria proliferate to form microcolonies and begin synthesizing an extracellular polymeric substance (EPS) matrix. In the Enterobacteriaceae family, this matrix is largely composed of exopolysaccharides such as poly-β-1,6-N-acetyl-D-glucosamine (PNAG) in the case of *Y. ruckeri* [7]. This extracellular matrix provides essential structural stability, protection against environmental stressors, and mechanical cohesion. Progression through these stages is tightly regulated by intracellular signaling molecules, most notably the second messenger cyclic diguanylate monophosphate (c-di-GMP). Elevated intracellular levels of c-di-GMP typically inhibit motility and promote the biosynthesis of matrix components, driving biofilm maturation, whereas reduced c-di-GMP levels trigger biofilm dispersal, allowing bacteria to reacquire motility and colonize new environments.

The substantial metabolic cost associated with transitioning between planktonic and sessile states requires stringent regulatory control to orchestrate a broad array of genes. This multi-stage developmental cycle is highly coordinated and driven by the precise reprogramming of gene expression, involving both transcriptional and post-transcriptional factors. However, the molecular mechanisms governing biofilm development and maintenance in fish pathogens like *Y. ruckeri* remain poorly understood. Previous studies have begun to identify structural and regulatory determinants of biofilm formation in *Y. ruckeri*. Early work demonstrated that isolates recovered from aquaculture environments can adhere to solid surfaces and linked this capacity to flagellum-mediated motility [5]. More recently, the surface-exposed inverse autotransporters YrInv and YrIlm were shown to promote biofilm formation on abiotic substrates and to contribute differentially to microcolony interaction and formation, respectively [10]. At the regulatory level, the two iron-responsive sRNAs RyhB-1 and RyhB-2, which are repressed by the global transcriptional regulator Fur and stabilized by the Hfq chaperone, exert differential effects on motility and biofilm formation [11]. Furthermore, Hfq and the Hfq-dependent sRNAs RprA, ArcZ, and RybB modulate motility, biofilm architecture, extracellular matrix-associated phenotypes, as well as the expression of genes related to the bacterial envelope and c-di-GMP metabolism [7].

Among the global transcriptional regulators integrating these complex biological processes is the Ferric Uptake Regulator (Fur). Classically defined as a metal-dependent DNA-binding protein, Fur maintains intracellular iron homeostasis by repressing iron acquisition systems under iron-replete conditions. However, Fur’s regulatory footprint extends far beyond its direct control of iron-acquisition systems. Fur influences broader physiological and virulence-associated networks, either directly or through iron-responsive regulatory circuits. Emerging evidence indicates that Fur functions globally and can modulate gene expression independently of iron availability. Specifically, the iron-free conformation of the protein (Apo-Fur) is structurally active and capable of binding distinct promoter sequences to regulate complex metabolic and virulence networks [12]. Furthermore, Fur exhibits extensive pleiotropic effects within human pathogens of the genus *Yersinia*, controlling virulence networks, oxidative stress responses, and biofilm formation regardless of iron availability [13].

The specific role of Fur in biofilm development is highly complex and species-dependent. In *Y. pestis*, Fur directly represses biofilm formation by downregulating the expression of a gene encoding the diguanylate cyclase HmsT, thereby reducing c-di-GMP production and biofilm formation [14]. Fur also represses the RyhB homologs, which positively influence *hmsT*, *hmsHFRS* expression, and PNAG production, establishing an additional indirect regulatory layer [15]. Likewise, in *Vibrio cholerae*, Fur has been shown to repress biofilm formation by negatively regulating the expression of the *vpsU* and *vpsA-K* genes, which are involved in EPS biosynthesis, as well as *cdgD*, which encodes a diguanylate cyclase [16]. Moreover, Fur activates the expression of the *vieSAB* operon, encoding the phosphodiesterase VieA that degrades c-di-GMP, thereby suppressing biofilm formation via an iron-independent mechanism [16]. Conversely, in other species such as *Edwardsiella piscicida* and *V. splendidus*, Fur has been described as essential for the positive activation of motility and structural matrix components [17,18].

Despite the recognized importance of Fur in bacterial physiology, its contribution to biofilm development in *Y. ruckeri* has not been experimentally established, and it is unclear how loss of Fur affects the temporal progression and three-dimensional organization of *Y. ruckeri* biofilms and which transcriptional programs accompany this phenotype. To address this knowledge gap, the present study investigated the role of the global transcriptional regulator Fur in the biofilm formation process of *Y. ruckeri*. The experimental strategy of this study combined a comprehensive phenotypic and structural characterization of a specifically designed *fur* deletion mutant to compare it with its wild-type counterpart under biofilm-forming conditions. This comparative assessment focused on key biofilm-associated traits, including flagellar synthesis, motility, and the architectural organization of biofilms, utilizing scanning electron and confocal microscopy techniques. Furthermore, to determine the underlying molecular pathways or factors governed by this regulator, global transcriptomic profiling was then used to identify Fur-dependent gene-expression changes at matched chronological time points selected to represent early, intermediate, and late phases of the wild-type biofilm-development trajectory. Our findings identify Fur as an important contributor to mature biofilm formation and three-dimensional organization in *Y. ruckeri* and provide a framework for investigating the underlying Fur-dependent regulatory network.

## 2. Results

### 2.1. Deletion of fur Reduces Motility and Flagellar Abundance in Y. ruckeri

**Figure 1:**
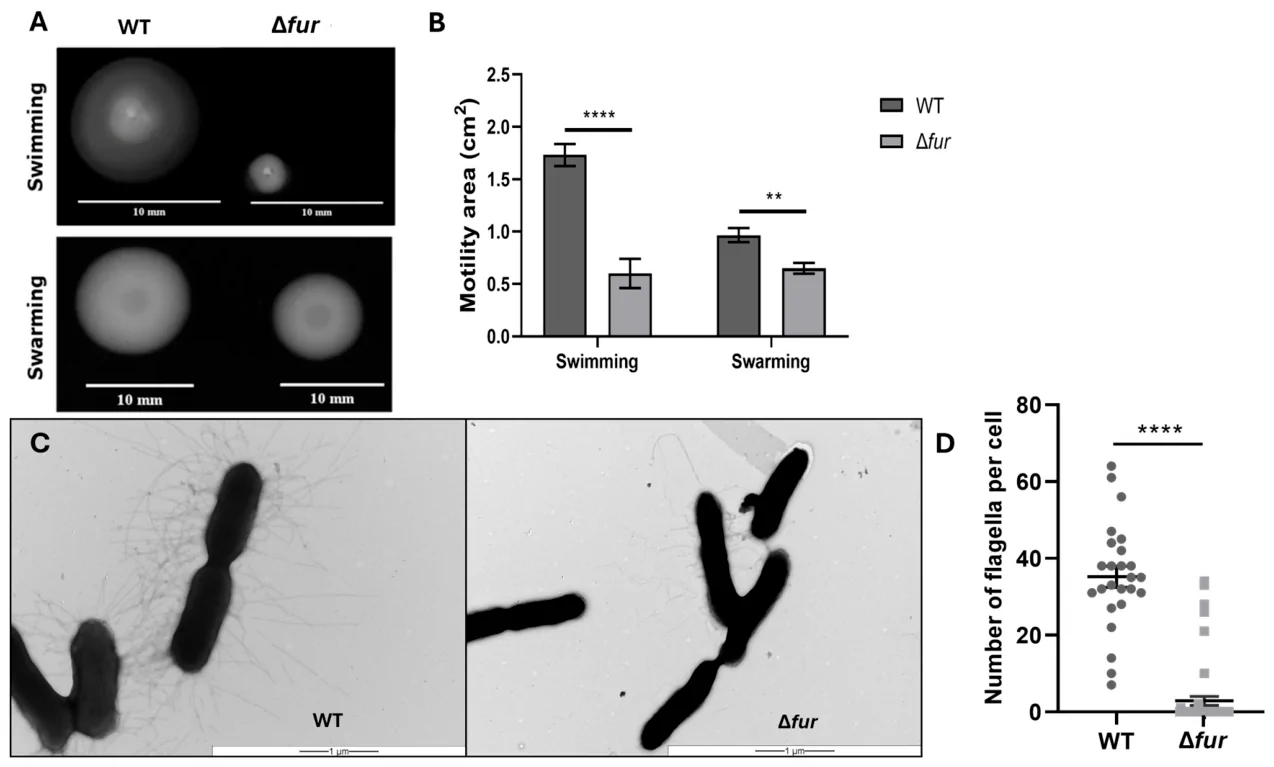
Assessment of motility and flagellar structure in wild-type (WT) and ∆*fur* strains of *Y. ruckeri*. (**A**) Swimming and swarming motility assays were performed by inoculating 1 μL of overnight cultures onto plates containing 0.3% (*w*/*v*) and 0.5% (*w*/*v*) agar, respectively. (**B**) Bar graph showing motility areas calculated from migration zone diameters. Data are presented as mean ± standard error (*n* = 9). Prior to statistical analysis, data normality was assessed independently for each group using the Shapiro–Wilk test, and homogeneity of variances was confirmed using Levene’s test. Statistical analysis was performed using two-way ANOVA followed by Tukey’s HSD post hoc test for pairwise comparisons (** *p* < 0.01; **** *p* < 0.0001). (**C**) Representative TEM micrographs of exponentially growing cells (OD_600_ = 0.5) to visualize the presence and distribution of flagellar appendages. (**D**) Box plot representing the number of flagella per cell, based on the analysis of ~50 individual bacteria. Given the discrete, count-type nature of flagella enumeration data, the nonparametric Mann–Whitney U test was applied (**** *p* < 0.0001).

Flagellum-dependent motility is essential for surface colonization and early biofilm development in *Y. ruckeri*. To determine whether *fur* deletion (Δ*fur*) affects motility in *Y. ruckeri*, swimming and swarming assays were performed on semisolid media. Overnight cultures of the wild-type and Δ*fur* strains were inoculated onto TSA plates containing 0.3% or 0.5% agar, respectively, and incubated at 26 °C for 24 h. Representative images of the resulting motility halos are shown in Figure 1A. Differences in migration zone diameters were observed between the two strains, with the Δ*fur* strain displaying reduced motility compared to the wild-type in both swimming and swarming assays. Figure 1B shows the average motility areas calculated from nine biological replicates. The results consistently revealed a 70% and 40% decrease in swimming and swarming motility, respectively, in the Δ*fur* strain. Expression of *fur* in the Δ*fur* background restored both swimming and swarming motility to levels comparable to those of the wild-type strain, supporting that the phenotype was associated with deletion of *fur* (Figure S1).

To determine whether the differences observed in motility assays were associated with alterations in flagellar structure, transmission electron microscopy (TEM) was performed. Representative TEM micrographs showed numerous filamentous surface structures consistent with flagella in wild-type cells, whereas such structures were less abundant in the Δ*fur* preparation. Quantification confirmed a lower number of putative flagellar filaments per cell in the mutant (Figure 1C,D).

### 2.2. Loss of Fur Impairs Late-Stage Biofilm Accumulation and Organization in Y. ruckeri

**Figure 2:**
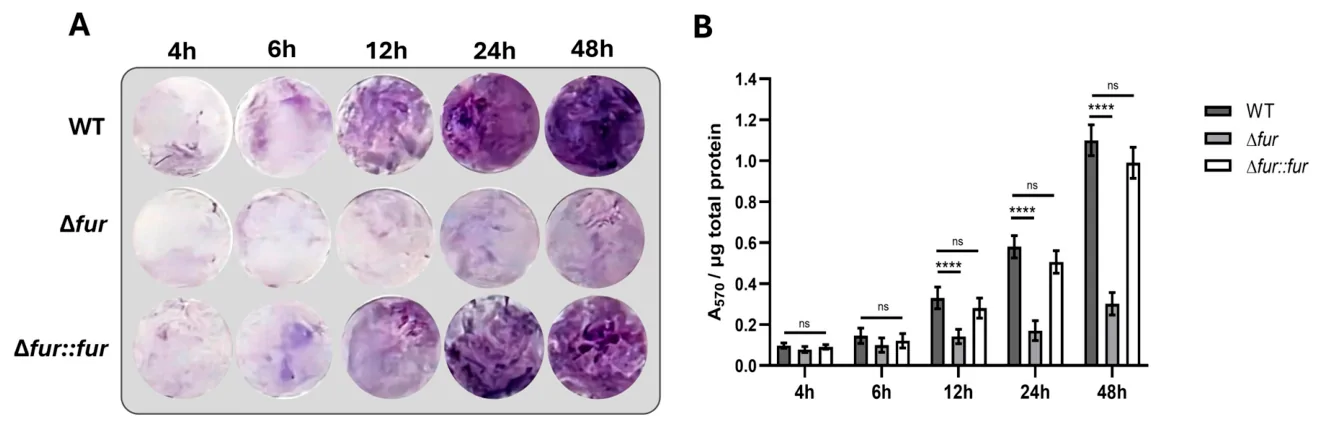
Crystal violet biofilm assay in wild-type (WT) and ∆*fur* strains of *Y. ruckeri*. (**A**) Biofilm formation assessed at 4, 6, 12, 24, and 48 h post-inoculation. Coverslips were stained with 1% (*v*/*v*) crystal violet, and images were captured with a gel documentation system. (**B**) Biofilm quantification based on absorbance at 570 nm following solubilization of crystal violet with 33% (*v*/*v*) acetic acid. Values were normalized according to total protein obtained from adhered cells recovered by low-frequency sonication. Data represent the mean ± standard deviation of three independent replicates (*n* = 3). Data normality was assessed independently for each group using the Shapiro–Wilk test, and homogeneity of variances was confirmed using Levene’s test. Statistical analysis was performed using two-way ANOVA followed by Tukey’s HSD post hoc test for pairwise comparisons. No statistically significant differences were detected at 4 and 6 h (ns: *p* ≥ 0.05). Statistically significant reductions in biofilm-associated biomass were observed from 12 h onwards (**** *p* < 0.0001).

To investigate the impact of Fur on total biofilm-associated biomass in *Y. ruckeri*, crystal violet (CV) staining was used as an estimate of total surface-associated biomass, including bacterial cells and extracellular material. Wild-type and Δ*fur* strains were cultured on silicate slides for 4, 6, 12, 24, and 48 h. No statistically significant differences were detected between the wild-type and Δ*fur* strains at 4 and 6 h. At 12, 24 and 48 h, the mutant showed trends toward lower CV retention, which were statistically significant, indicating a reduction in biofilm-associated biomass (Figure 2A,B). On the other hand, the complementation of the Δ*fur* strain restored the phenotype to a similar extent to that observed in the wild-type strain, confirming that the difference in biofilm formation observed was due to the deletion of *fur*. Because the Δ*fur* strain showed delayed growth (Figure S2), differences in proliferation may contribute to the biofilm phenotype. Nevertheless, the pronounced reduction in retained biomass and structural organization at later times suggests that growth impairment alone may not fully account for the observed phenotype.

**Figure 3:**
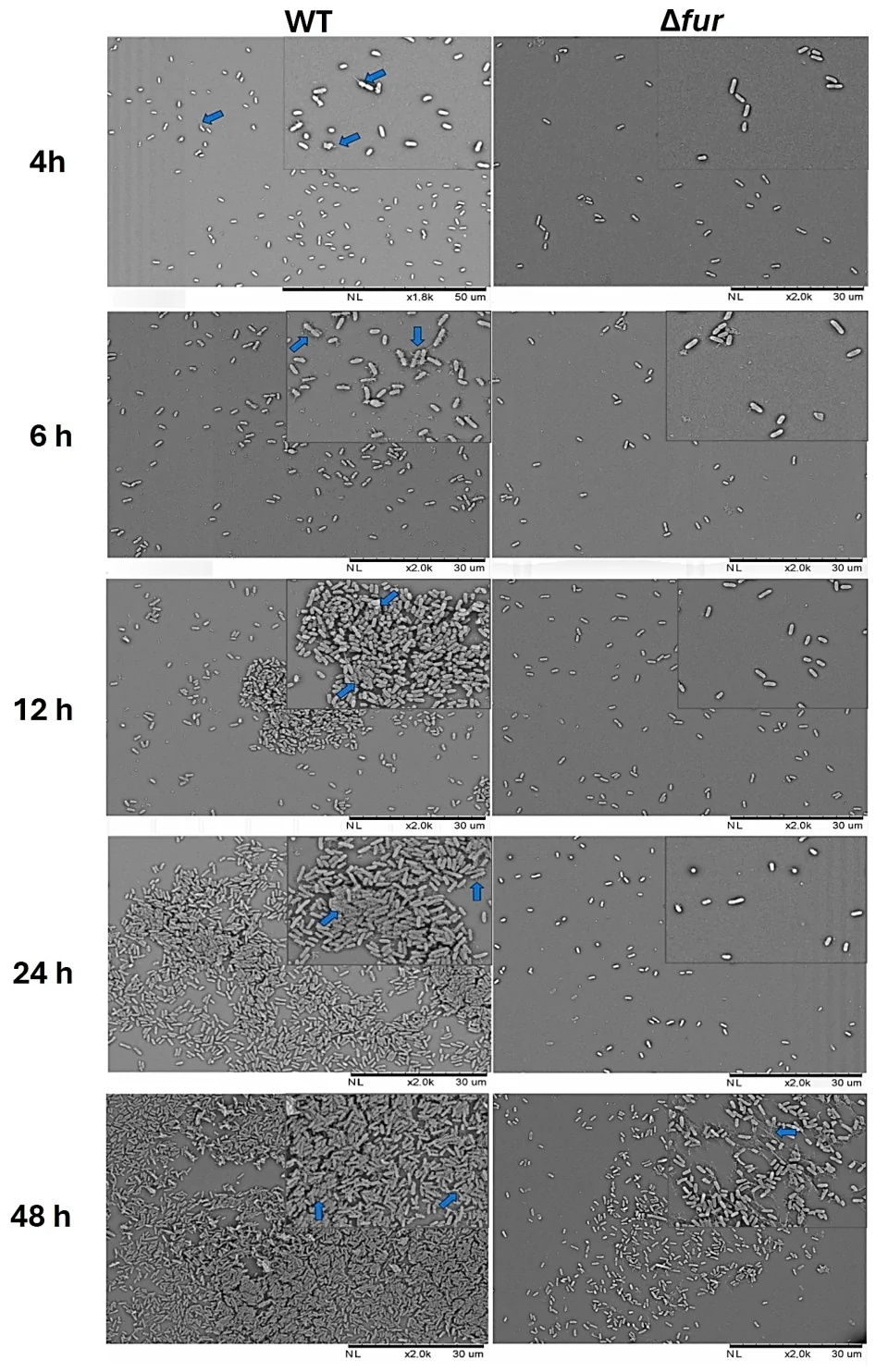
Structural analysis of *Y. ruckeri* biofilms of wild-type (WT) and ∆*fur* strains by SEM. Biofilms formed by the WT (left panels) and ∆*fur* (right panel) strains were grown on silicate covers and incubated for 4, 6, 12, 24, and 48 h. At each time point, samples were fixed with 2.5% glutaraldehyde for 24 h, subsequently processed by critical point drying, and sputter-coated with gold. Images were acquired using a Hitachi TM3000 scanning electron microscope. Representative micrographs are shown for each strain and time point. Arrows indicate representative filamentous extracellular structures.

To further evaluate biofilm architecture by analyzing both cellular morphology and spatial organization, scanning electron microscopy (SEM) was performed. Representative SEM images are shown in Figure 3. At 4 and 6 h, representative SEM fields showed sparse surface-associated cells in both strains, with no obvious qualitative difference in early attachment. However, filamentous structures resembling appendages or components of the extracellular matrix (indicated by blue arrows) were visible only in the wild-type. From 12 h onward, larger and more frequent cell aggregates were evident in representative wild-type fields, whereas Δ*fur* cells remained comparatively dispersed and formed less developed clusters. At 24 and 48 h, this qualitative difference in cellular organization became more pronounced.

**Figure 4:**
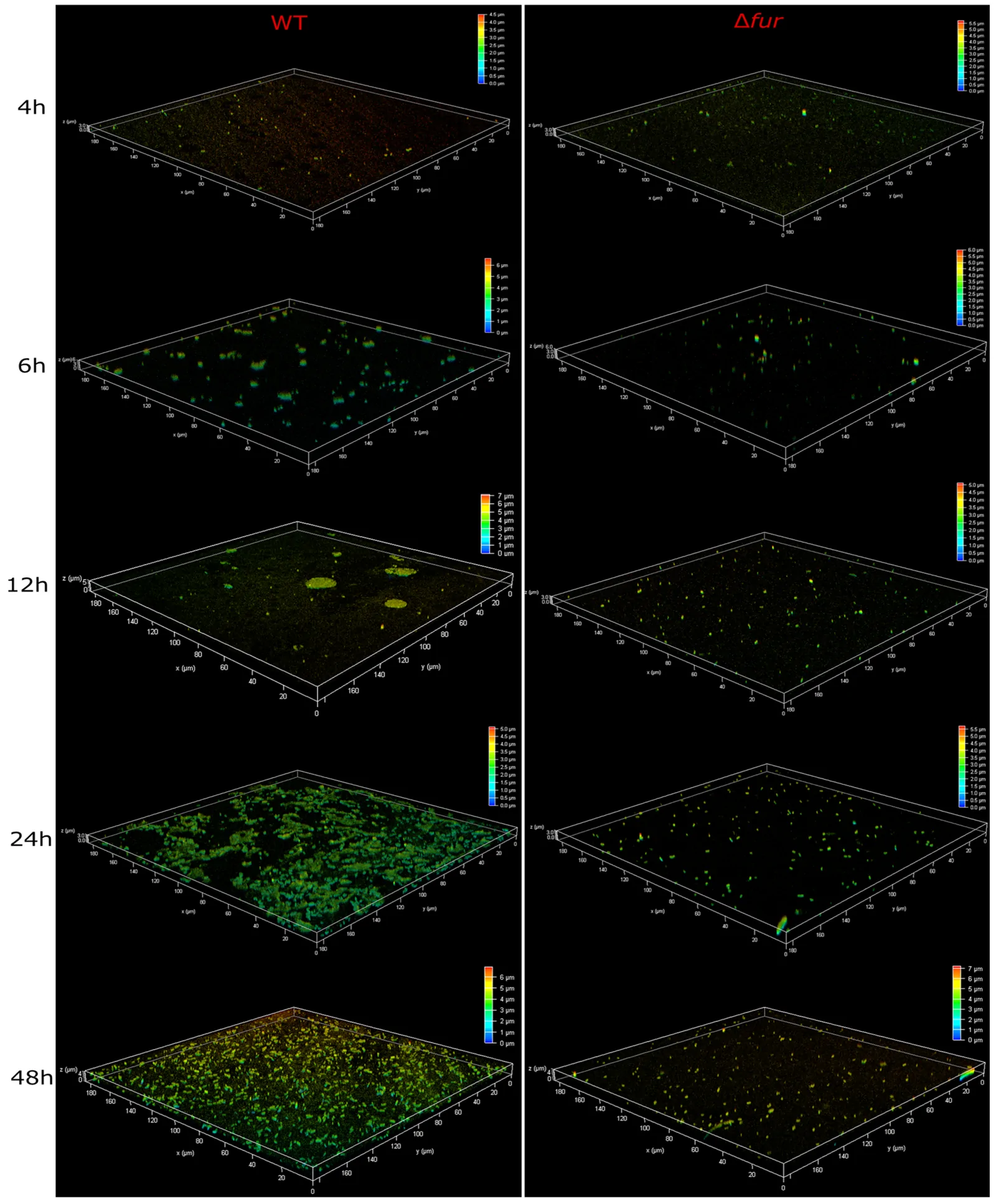
Confocal microscopy analysis of *Y. ruckeri* wild-type (WT) and Δ*fur* biofilms. *Y. ruckeri* strains were previously transformed with the pDiGc plasmid to visualize the cells. Wild-type (WT) and Δ*fur* strains were grown on silicate covers without shaking at 22 °C for the indicated times (4, 6, 12, 24, and 48 h). After incubation, covers were washed and fixed with 4% paraformaldehyde. Samples were visualized on a Leica TCS SP8 confocal microscope at 6000× magnification, and images were reconstructed using LAS X software 3.1.5. The color scale (heatmap) in each panel represents the depth (*Z*-axis) of the biofilm in micrometers (µm). Representative images from each time point are shown (*n* = 3).

To complement the structural analysis with a three-dimensional view of biofilm architecture, laser scanning confocal microscopy (LSCM) was performed. Representative three-dimensional reconstructions showed a progressive increase in surface coverage and cellular aggregation in the wild-type strain from 12 to 48 h. In comparison, Δ*fur* biofilms displayed lower apparent coverage and less developed three-dimensional aggregates, particularly from 12 h onward (Figure 4).

**Figure 5:**
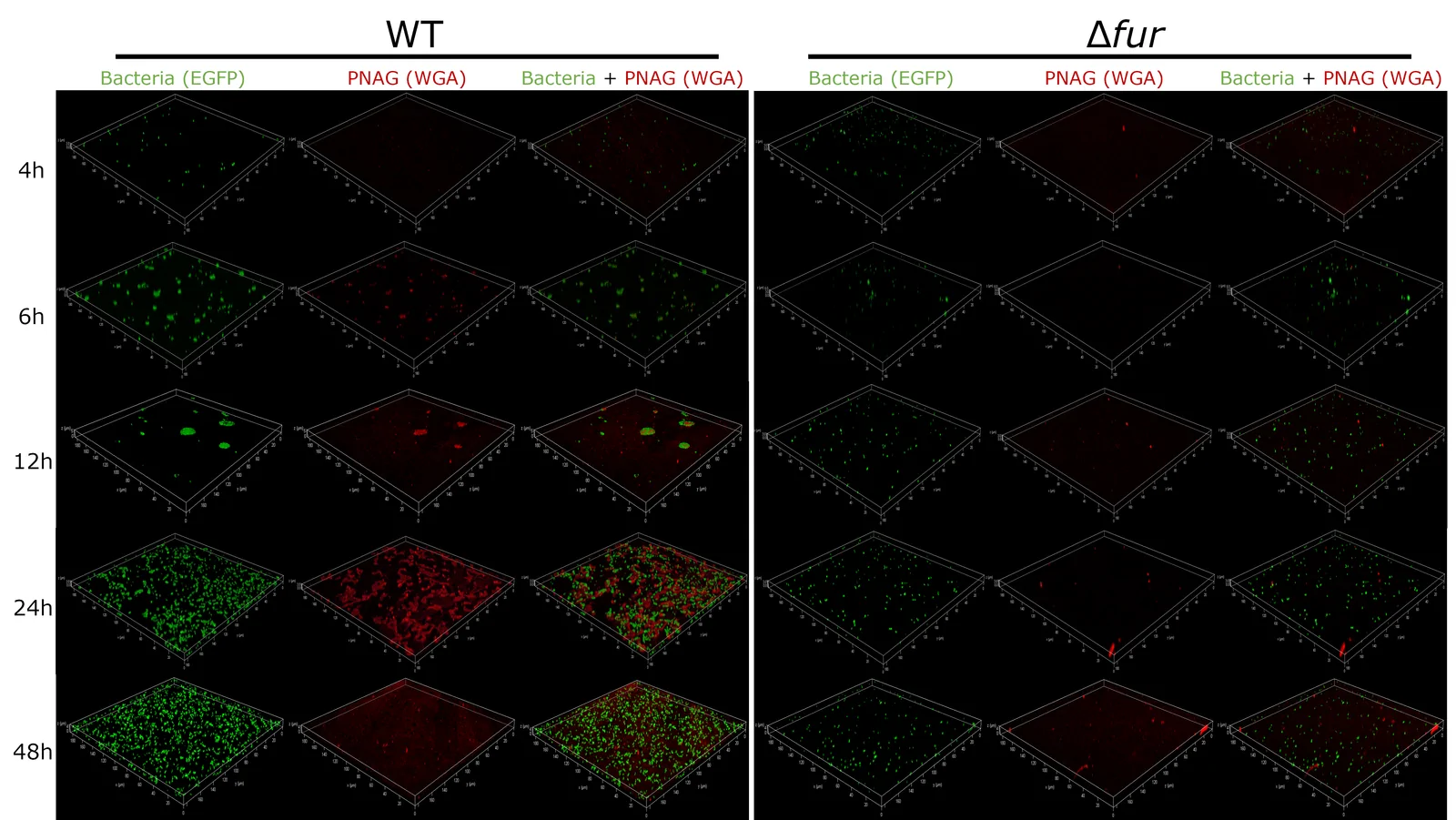
Confocal microscopy analysis of N-acetyl-D-glucosamine (GlcNAc) Presence in *Y. ruckeri* wild-type (WT) and Δ*fur* biofilms. *Y. ruckeri* strains were previously transformed with the pDiGc plasmid to visualize the cells. Wild-type (WT) and Δ*fur* strains were grown on silicate covers without shaking at 22 °C for the indicated times. After incubation, covers were washed, stained with Alexa Fluor 647–conjugated wheat germ agglutinin (WGA), and fixed in 4% (*v*/*v*) paraformaldehyde. In each panel, the left column (GFP) shows green-labeled bacteria, the middle column shows WGA-Alexa Fluor 647-reactive material and is interpreted as a proxy for GlcNAc-containing matrix components, and the right column (merge) shows the overlay of both channels. Samples were imaged using a Leica TCS SP8 confocal microscope at 6000× magnification, and images were reconstructed with LAS X software. Representative images from each time point are shown (*n* = 3).

Since poly-β-1,6-N-acetyl-D-glucosamine (PNAG) is a component of the extracellular matrix in *Y. ruckeri* biofilms, we examined its production in the biofilms of both Δ*fur* and wild-type strains. To visualize WGA-reactive N-acetylglucosamine (GlcNAc)-containing material associated with the biofilm matrix, samples were stained with Alexa Fluor 647-conjugated wheat germ agglutinin. In wild-type biofilms, the WGA-associated signal overlapped spatially with GFP-labeled cellular aggregates, particularly at 12 and 24 h. In contrast, from 12 h onward, Δ*fur* biofilms showed lower total GFP-labeled biomass and lower WGA-associated fluorescence than wild-type biofilms (Figure 5). These observations indicate reduced accumulation of WGA-reactive matrix-associated material at the biofilm level in the ∆*fur* strain.

Together, the CV, SEM, and LCSM analyses indicate that *fur* deletion impairs the progression of *Y. ruckeri* toward dense, spatially organized biofilms and is associated with reduced accumulation of matrix-associated GlcNAc.

### 2.3. Analysis of c-di-GMP Levels in Wild-Type and Δfur Biofilms of Y. ruckeri

**Figure 6:**
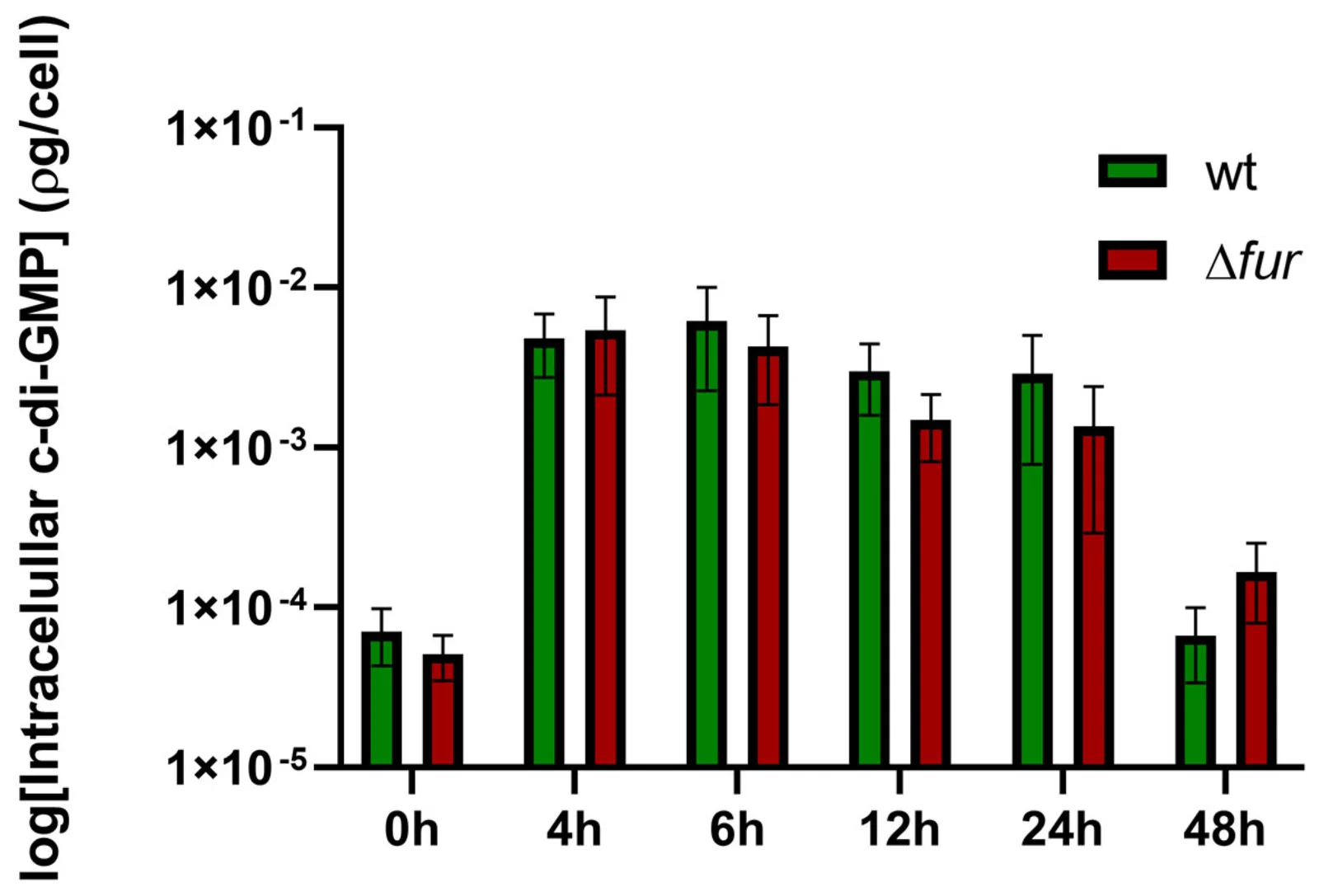
Intracellular c-di-GMP levels in *Y. ruckeri* wild-type (WT) and Δ*fur* biofilms. Wild-type (WT) and Δ*fur* strains were grown without shaking at 22 °C on silicate covers for the indicated times of biofilm formation (*X* axis). Adherent cells were recovered by low-frequency sonication, resuspended in phosphate buffer, and subjected to c-di-GMP. Results represent three independent replicates. Data represent the means ± standard deviations (*n* = 3). Statistical analysis was performed using two-way ANOVA. No statistically significant differences were detected between wild-type (wt) and Δ*fur* strains at any time point.

Given that biofilm formation is tightly regulated by c-di-GMP, the intracellular levels of this second messenger were measured over the course of biofilm development in both strains. For this analysis, bacterial cells were recovered from biofilms to quantify c-di-GMP levels by ELISA, as previously described [7]. At the initial time (0 h), corresponding to planktonic cells, the intracellular c-di-GMP levels in both strains were lower than those recovered from biofilms at 4, 6, 12, and 24 h (Figure 6). At 48 h, a reduction in c-di-GMP levels of both strains was observed as compared to earlier times. However, no statistically significant differences in c-di-GMP concentrations were detected between the two strains at any of the time points analyzed (Figure 6). These data do not support a major effect of *fur* deletion on the bulk intracellular c-di-GMP pool of *Y. ruckeri* biofilms under the conditions tested.

### 2.4. Deletion of fur Is Associated with Time-Dependent Transcriptional Changes During Y. ruckeri Biofilm Development

The transition from the planktonic state to a mature biofilm involves a precise reprogramming of bacterial gene expression, which modulates key functions such as motility, adhesion, extracellular matrix production, and, ultimately, cell dispersion. In this context, a global transcriptomic analysis via RNA-seq was conducted on the wild-type and ∆*fur* strains of *Y. ruckeri*. Based on our preliminary phenotypic characterization, three time points were chosen to capture biologically and phenotypically distinct phases of the biofilm developmental trajectory, each serving a specific experimental purpose: (i) 6 h, the early surface attachment phase, in which cells of both strains are present on the surface but bulk biomass accumulation is not yet significantly different between genotypes; this time point is particularly informative for identifying the initial Fur-dependent transcriptional decisions activated upon surface contact, before pronounced phenotypic divergence occurs; (ii) 12 h, the intermediate transition phase, at which statistically significant, genotype-dependent differences in biofilm biomass first emerge; transcriptomic profiling at this stage captures the transition from early attachment to structured microcolony development and the regulatory programs that underlie this shift; and (iii) 48 h, the late-stage mature biofilm, at which the most pronounced architectural, structural, and biomass differences between wild-type and Δ*fur* strains are observed across crystal violet, SEM, and confocal microscopy analyses.

Principal component analysis of the transcriptomic profiles confirmed a clear separation between the wild-type and Δ*fur* strains, as well as between biofilm stages, with high reproducibility among biological replicates.

**Figure 7:**
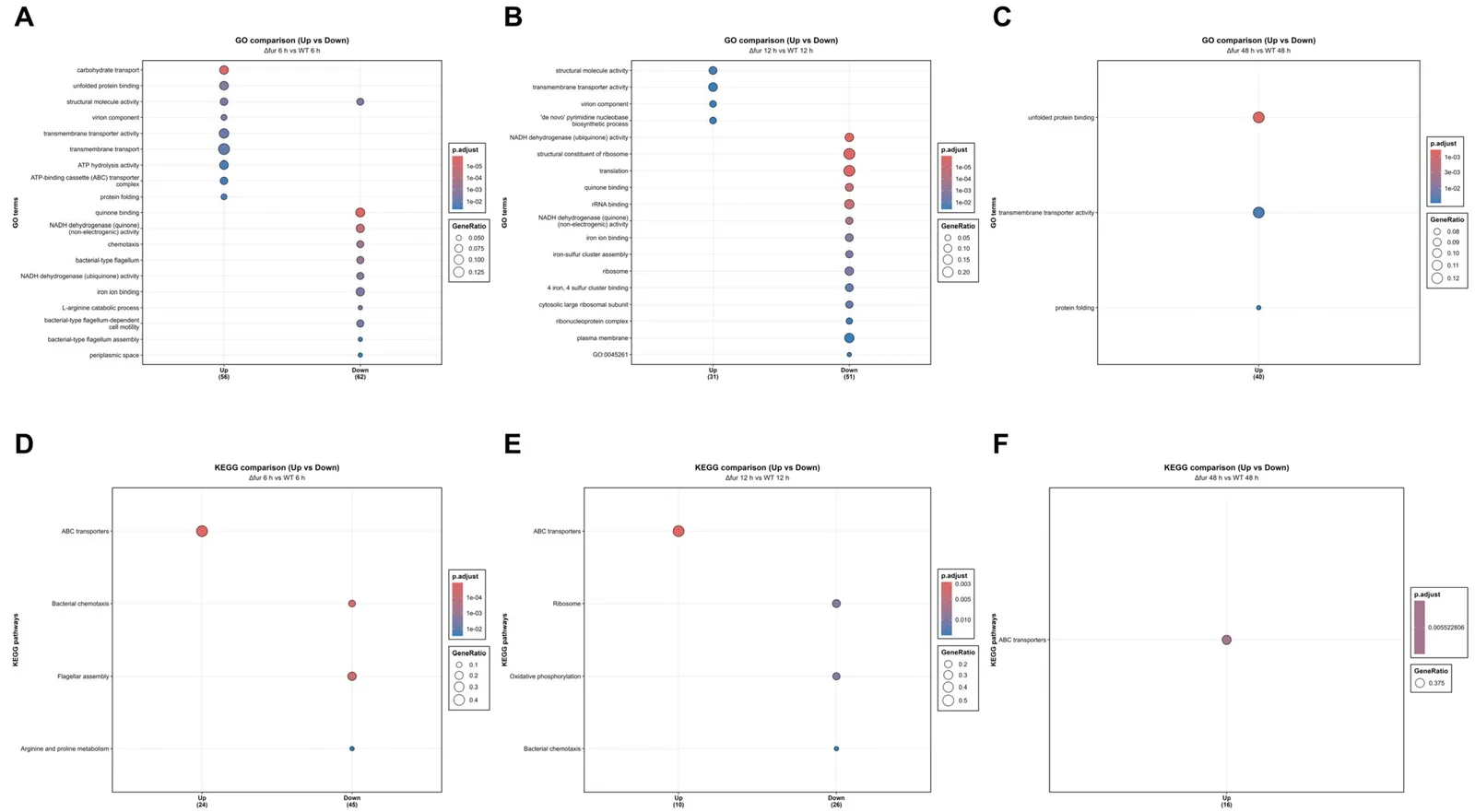
GO and KEGG enrichment comparison of up- and down-regulated genes in the Δ*fur* strain during biofilm formation. Dot plots comparing enriched Gene Ontology terms (**A**–**C**) and KEGG pathways (**D**–**F**) between up- and down-regulated genes of the Δ*fur* strain relative to the wild-type at 6 h (**A**,**D**), 12 h (**B**,**E**), and 48 h (**C**,**F**). Within each panel, terms are split by regulation direction (Up vs. Down); the number of genes in each set is indicated in parentheses. Dot size represents the GeneRatio and color represents the adjusted *p*-value.

To highlight the functional differences between the wild-type and Δ*fur* strains at each stage, GO and KEGG enrichment analyses were performed by contrasting the up- and down-regulated genes of the mutant at every time point (Figure 7; the corresponding gene-concept networks are shown in Figure S3). Across all three stages, the up-regulated genes in the absence of Fur were consistently enriched in transmembrane transport and ATP-binding cassette (ABC) transporter functions associated with iron acquisition (Figure 7A–C, Up), a pattern also captured at the pathway level as a sustained enrichment of ABC transporters (Figure 7D–F, Up). This reflects the expected de-repression of iron-uptake systems in the absence of the master iron-responsive regulator.

The down-regulated genes, in contrast, showed a stage-dependent pattern. At the early stage (6 h), the most repressed functions were bacterial chemotaxis, flagellar assembly, and flagellum-dependent motility (Figure 7A,D), consistent with the reduced motility phenotype previously observed in the Δ*fur* strain. At 12 h, repression shifted toward translation and ribosome biogenesis, oxidative phosphorylation, and iron–sulfur cluster assembly (Figure 7B,E), indicating a broad down-modulation of the biosynthetic and respiratory machinery. At the late stage (48 h), few functions remained significantly down-regulated, while the up-regulated set became enriched in protein folding and unfolded-protein binding (Figure 7C), pointing to an increased chaperone-associated stress response in the mutant strain.

**Figure 8:**
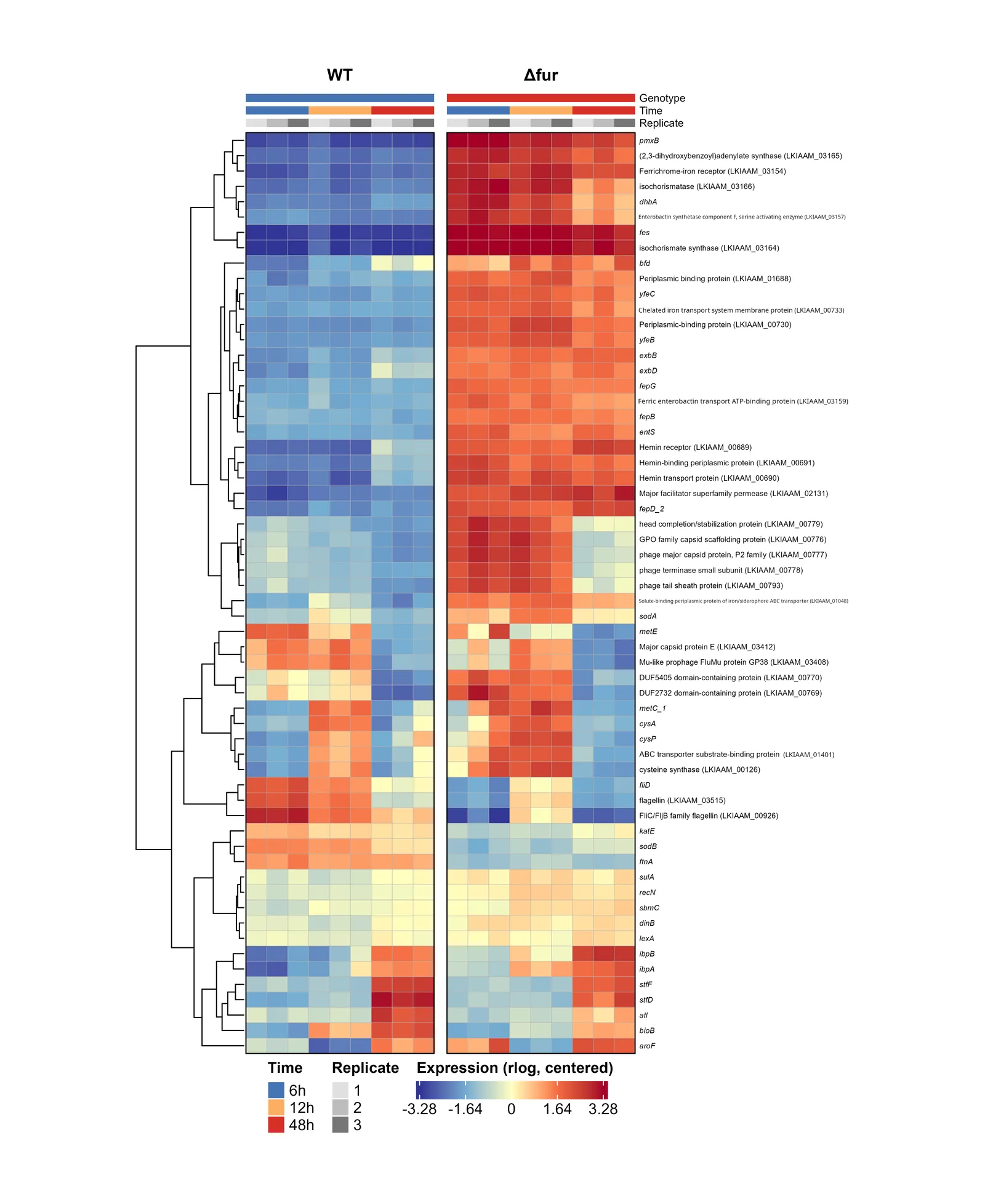
Expression heatmap of the 50 most variable genes together alongside a curated selection of biologically relevant targets involved in oxidative stress, the SOS response, and prophage induction, across biofilm formation in *Y. ruckeri* wild-type (WT) and Δ*fur* strains. Rows show regularized-log-transformed, row-centered expression. Columns are grouped by genotype (WT, **left**; Δ*fur*, **right**) and ordered by biofilm stage (6, 12 and 48 h) and biological replicate (1–3), as indicated by the top annotation bars. Selected genes relevant to the proposed regulatory model (e.g., *sodA*, *sodB*, *katE*, and *ftnA*, as well as the SOS genes *sulA*, *recN*, *dinB*, *sbmC*, *lexA*, and *aroF*) were included regardless of their variance ranking. The color scale represents row-centered expression (blue, low; red, high). Gene symbols are shown where available; otherwise, the product description and locus tag are given.

In addition, a heatmap was generated displaying the 50 genes with the greatest expression variability between the wild-type and Δ*fur* strains across biofilm formation, alongside a curated selection of biologically relevant targets involved in oxidative stress, the SOS response, and prophage induction (Figure 8). This targeted visualization strategy was employed because, although some of these stress-related genes do not exhibit the highest absolute fold-changes globally, their coordinated and temporal expression patterns provide crucial insights into the pleiotropic regulatory scope of Fur. Among the genes strongly up-regulated in the Δ*fur* strain, we identified those involved in siderophore biosynthesis, transport, and iron uptake. These include genes automatically annotated as *fes*, *entS*, *fepB*, *fepG*, and *dhbA*, which represent orthologs of the native *Y. ruckeri* ruckerbactin system (*ruc locus*) [19], together with the TonB-complex components *exbB* and *exbD*. Furthermore, the analysis revealed an up-regulation of the *yfeB* and *yfeC* iron/manganese ABC transporter components, as well as the bacterioferritin-associated ferredoxin, *bfd*.

Beyond iron homeostasis, the curated heatmap profiles highlight distinct adaptive stress-response dynamics. Specifically, the data show a strong and sustained up-regulation of the non-ribosomal peptide gene *pmxB*. Furthermore, SOS response genes, including *sulA*, *recN*, *dinB*, *sbmC*, and *lexA*, displayed a coordinated up-regulation in the Δ*fur* strain, specifically at the intermediate and late stages of biofilm formation. Conversely, the absence of Fur led to the down-regulation of flagellar components (*fliD* and *flgK*) and key oxidative stress-response genes (*sodB*, *katE*, and *ftnA*). Likewise, the *napABCD* operon, whose products constitute the periplasmic nitrate reductase complex active under the microaerophilic conditions found within a biofilm, was also down-regulated.

Interestingly, the targeted visualization also revealed the strong and temporally coordinated up-regulation of a resident prophage in the Δ*fur* strain. This induction included structural genes (major capsid, tail, and terminase components) and lytic effectors (a holin and an endolysin), which peaked particularly at the early and intermediate stages of biofilm development.

**Figure 9:**
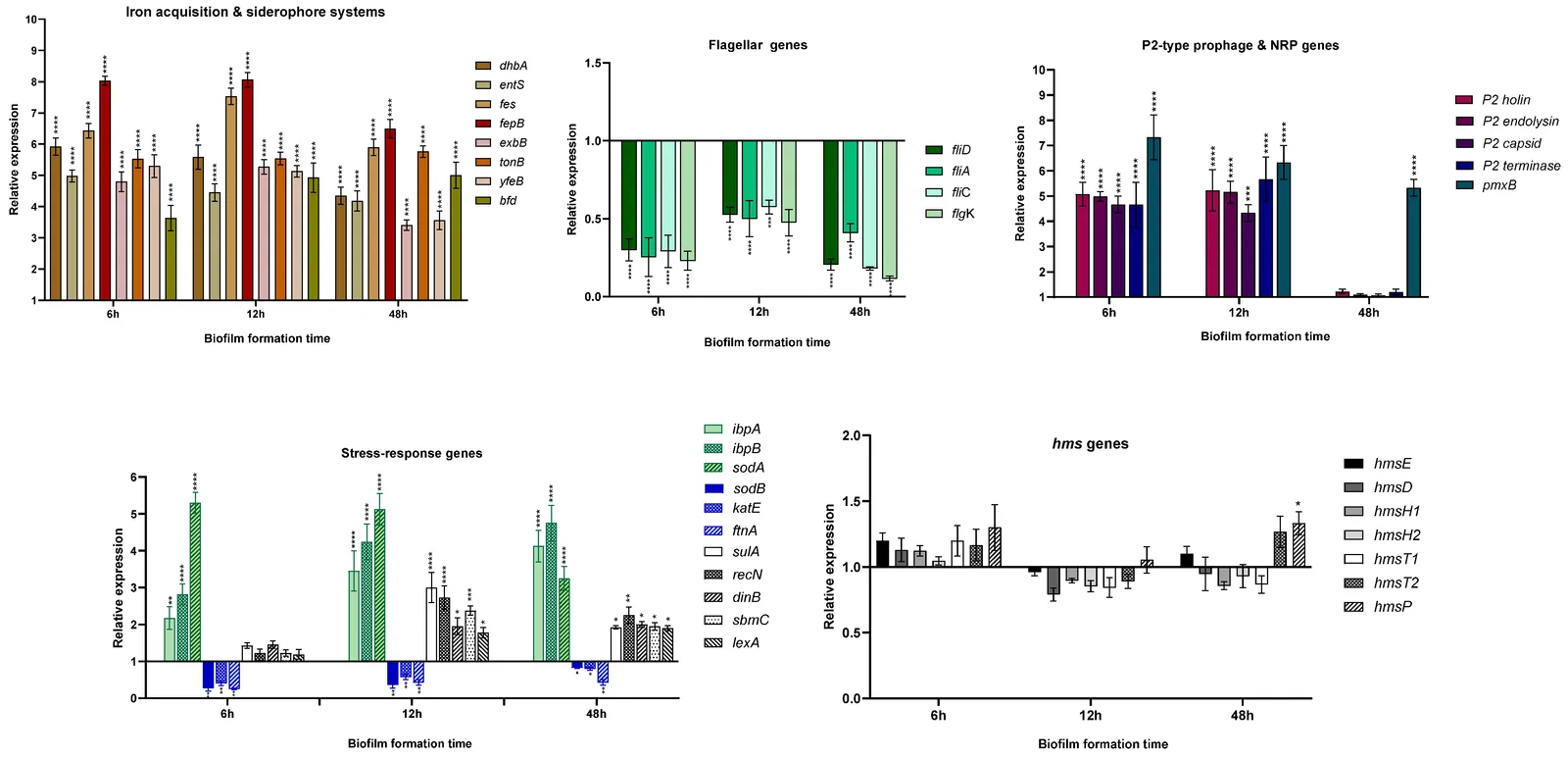
Relative gene expression analysis by RT-qPCR in the ∆*fur* strain of *Y. ruckeri* during biofilm formation. Wild-type (WT) and Δ*fur* strains were grown without shaking at 22 °C on covers for the indicated times. Adherent cells were recovered by low-frequency sonication and subjected to RNA extraction. Gene expression was quantified by RT-qPCR and is shown relative to WT. Statistical comparisons between WT and Δ*fur* at each time point were performed using Student’s *t*-test, following confirmation of data normality (Shapiro–Wilk test) and homogeneity of variances (Levene’s test) for each group independently. Asterisks represent statistically significant differences with respect to the WT and are indicated as *p* = 0.025 (*hms*), *p* < 0.05 (*), *p* < 0.01 (**), *p* < 0.001 (***), *p* < 0.0001 (****), and ns = *p ≥* 0.05. Data represent the means ± standard deviations (*n* = 3).

Finally, using RT-qPCR, we validated the expression of representative genes associated with flagellar synthesis, siderophore-mediated iron acquisition, stress response, pro-phage, and specific amino acid biosynthesis pathways. Among the genes analyzed, we also included the Hms system involved in c-di-GMP and PNAG metabolism [14]. As shown in Figure 9, the up-regulation of genes related to iron acquisition, SOS stress response, pro-phage synthesis, and non-ribosomal peptides observed in the absence of Fur was confirmed, consistent with the RNA-seq results. For its part, the down-regulation of genes for flagellar synthesis and oxidative stress response was also validated, while no differences were detected for the *hms* genes. Complementation of the Δ*fur* strain restored transcript levels to those of the wild-type, confirming that the changes in gene expression were due to the deletion of the *fur* gene (Figure S4).

Together, the RNA-seq and RT-qPCR analyses identified Fur-dependent expression changes involving iron acquisition, flagellar functions, stress responses, and specific genes related to a resident prophage and a non-ribosomal peptide. In contrast, the analyzed *hms* transcripts and bulk intracellular c-di-GMP levels showed no detectable genotype-dependent differences under the conditions tested.

**Figure 10:**
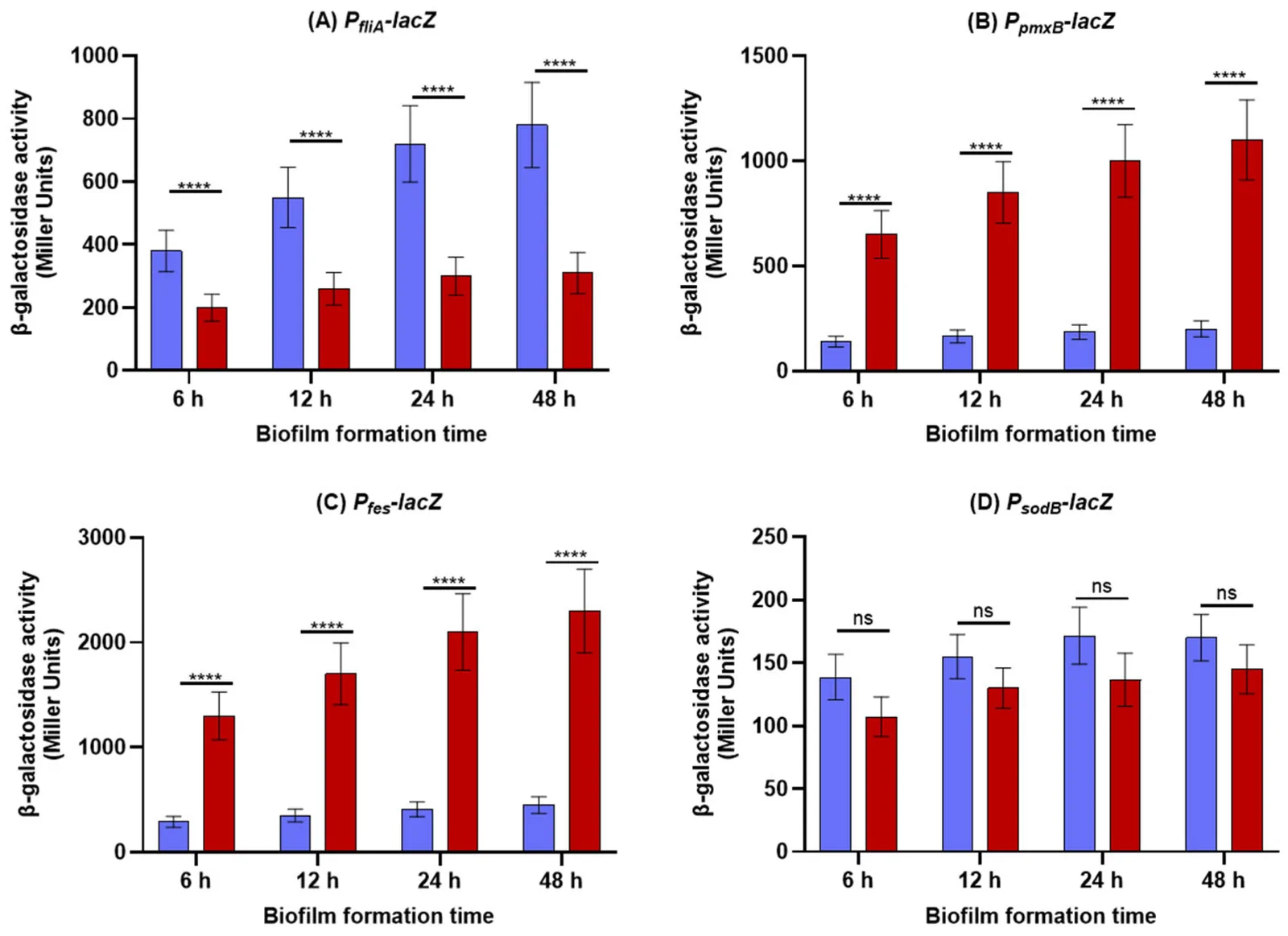
β-galactosidase activity from transcriptional reporter fusion assays in *Y. ruckeri* wild-type (WT, blue bars) and Δ*fur* (red bars) strains during biofilm formation. Bacteria were grown at 22 °C under static conditions for 6, 12, 24, and 48 h. Promoter activity was assessed for: (**A**) *PfliA-lacZ*, encoding the flagellar sigma factor; (**B**) *PpmxB-lacZ*, encoding a non-ribosomal peptide synthetase; (**C**) *Pfes-lacZ*, encoding the enterobactin esterase (ruckerbactin system); and (**D**) *PsodB-lacZ*, encoding the superoxide dismutase SodB. Bars represent mean β-galactosidase activity (Miller Units) ± SEM from three independent experiments (*n* = 3). Statistical analysis was performed using two-way ANOVA with Tukey’s HSD post hoc test to assess the overall genotype × time interaction, and Student’s *t*-test for pairwise comparisons between WT and Δ*fur* at each individual time point. Significance is indicated as: **** *p* < 0.0001; ns, not significant (*p* > 0.05).

To further validate initial RNA-seq and RT-qPCR data, and uncouple promoter dynamics from post-transcriptional mechanisms, we implement transcriptional reporter fusion assays. Transcriptional fusions were generated and assessed for five key Fur-dependent targets: flagellar gene *fliA*, non-ribosomal peptide gene *pmxB*, enterobactin gene *fes* (ruckerbactin system), and superoxide dismutase gene *sodB*. Results indicated that promoter activity for *fliA*, *pmxB*, and *fes* mirrored differential expression trends observed in global transcriptomic data, confirming Fur-dependent transcriptional regulation for these specific loci. Conversely, *sodB* promoter activity did not exhibit comparable modulation (Figure 10), indicating that *sodB* differential expression relies upon post-transcriptional regulation, as previously demonstrated [11].

## 3. Discussion

In human pathogens of the *Yersinia* genus like *Y. pestis*, as well as in the aquatic bacterium *V. cholerae*, Fur has been shown to function as a repressor of biofilm formation [14,16,20]. Although Fur-dependent regulation had previously been linked to motility and biofilm-associated phenotypes in *Y. ruckeri* through the iron-responsive sRNAs RyhB-1 and RyhB-2 [11], the consequences of directly deleting *fur* on biofilm development, three-dimensional organization, and biofilm-associated transcriptional programs had not been comprehensively examined. Here, we provide an integrated characterization of these Fur-dependent phenotypes.

### 3.1. The Role of Fur in the Motility of Y. ruckeri

The initial step of biofilm formation typically involves bacterial adhesion to a suitable surface, which represents a key stage in the development of a biofilm. In motile species such as *Y. ruckeri*, motility enables colonization of diverse surfaces and plays a crucial role in the initial attachment phase [21]. Regarding this physiological function, Fur has been shown to regulate motility in several bacterial species. For instance, in uropathogenic *Escherichia coli*, Fur represses motility by directly regulating *flhD*, which, along with *flhC*, forms the central transcriptional complex for flagellar biosynthesis [22], thereby affecting adhesion. In contrast, in other bacteria, Fur acts as a positive regulator of motility and flagellar synthesis. This has been described in *E. coli* AE17, a relevant pathogen in the poultry industry [23], where Fur directly activates *flhD* expression. Similar phenotypes have also been reported in *Helicobacter pylori* and *Chromobacterium violaceum*, where *fur* deletion negatively impacts motility [24,25].

In our study, the ∆*fur* strain of *Y. ruckeri* exhibited a ~70% reduction in swimming motility and a ~40% reduction in swarming motility (Figure 1A,B). In *H. pylori*, *fur* inactivation reduces motility without substantially altering flagellar number or length; instead, the phenotype has been associated with impaired flagellar motor switching [24]. In addition, the Δ*fur* phenotype observed here was accompanied by a marked reduction in the abundance of filamentous structures consistent with flagella, suggesting that distinct Fur-dependent mechanisms may operate in *Y. ruckeri*. These motility and structural phenotypes are in accordance with the down-regulation of key flagellar genes in the Δ*fur* strain, including the sigma factor *fliA*, flagellin *fliC*, filament-capping protein *fliD*, and the hook-associated protein *flgK* [26]. The expression of these genes was consistently repressed across all biofilm stages, together with the sigma factor *fliA* from the early stage (Figure 8 and Figure 9). Regarding *fliA* expression, the transcriptional reporter fusion assay confirmed Fur-dependent *fliA* transcription. In this context, it is important to mention that Fur-dependent positive modulation of motility occurs in other Gram-negative bacteria [24,25], and that direct interaction between Fur and the promoter of the flagellar master regulator *flhDC* has been established [23]. Therefore, it is plausible that Fur-dependent *fliA* activation relies upon FlhDC, representing evolutionarily conserved mechanisms coupling iron sensing to motility. To unequivocally determine whether this modulation constitutes a regulatory axis involving FlhDC within *Y. ruckeri*, future investigations must prioritize demonstrating physical interaction between Fur and the *flhDC* promoter region. Consequently, in vitro approaches, such as Electrophoretic Mobility Shift Assays (EMSA) utilizing purified Fur protein, or in vivo strategies including Chromatin Immunoprecipitation (ChIP), are required to yield definitive evidence of direct binding.

### 3.2. Fur Is Required for Multicellular Aggregation and Extracellular Architecture of Y. ruckeri Biofilms

Importantly, the Fur-dependent phenotype was most evident during later biofilm development rather than during the earliest surface-associated phase (Figure 2, Figure 3, Figure 4 and Figure 5). It is plausible that the phenotypes observed in the biofilm analyses conducted in this study reflect a combination of uncontrolled processes that go beyond motility alone, e.g., proliferation, adhesion, synthesis and secretion of extracellular matrix components, and cell dispersion. Representative microscopy fields showed surface-associated cells of both strains at early times, whereas pronounced differences emerged as the wild-type population progressed toward larger aggregates and a more organized three-dimensional biofilm. Thus, Fur appears to be particularly important for the progression toward mature biofilm organization, although quantitative analysis of early attachment would be required to determine whether more subtle differences are also present at this stage. Importantly, flagella are not only motility organelles but have also been reported to function as adhesins in several pathogens such as *E. coli* and *Pseudomonas aeruginosa* [27,28]. In this context, it is reasonable to consider that the diminished biofilm-forming capacity observed in the absence of Fur is a consequence, at least in part, of the altered flagellar structures observed in this strain. Nevertheless, the temporal separation between the motility defect and the pronounced impairment in mature biofilm organization suggests that altered flagellation is unlikely to represent the sole mechanism involved. Additional Fur-dependent processes affecting growth, cell–cell interactions, and matrix accumulation may also contribute.

To further investigate the underlying causes of the altered biofilm formation, we analyzed the structural characteristics of biofilms via microscopy analyses. Representative SEM images qualitatively showed denser cellular organization and more prominent filamentous extracellular structures in wild-type than in Δ*fur* biofilms, particularly at later times. Because these structural features were not quantitatively analyzed or molecularly identified, they should be interpreted as morphological associations rather than as defined matrix components (Figure 3).

It is important to mention that the relationship between motility and cell aggregation is not necessarily linear and may vary among different species. For instance, in *P. aeruginosa*, loss of motility in a *flgE*-deficient strain revealed reduced initial adhesion but enhanced formation of microcolony aggregates [29]. In the case of *Y. ruckeri*, our findings indicate that, beyond their role in surface colonization, flagellin subunits may contribute directly to biofilm architecture, potentially through interactions with extracellular polymeric substances that support matrix stability. In accordance with these findings and observations, *fliC* expression is significantly down-regulated in the *Y. ruckeri* ∆*fur* strain (Figure 9).

SEM imaging also revealed that the ∆*fur* strain exhibits a marked reduction in microcolonies, resulting in a less compact and organized biofilm structure compared to the wild-type strain. This observation suggests that the absence of Fur significantly impairs the bacterium’s ability to produce or regulate matrix components or interferes with the mechanical interactions that promote microcolony cohesion. If Fur functions as a positive regulator of key genes involved in matrix synthesis and the formation of adhesive structures (e.g., flagella, fimbriae, and EPS), its deletion may lead to a deficit in the production of these critical components, thereby compromising the stable formation of microcolonies and, consequently, the establishment of viable biofilms. Given the central role of EPS in biofilm architecture, particularly in mediating intercellular adhesion and providing mechanical support [30], the observed spatial disorganization in the ∆*fur* strain suggests a functional disruption in matrix assembly. In fact, confocal microscopy analysis revealed that the ∆*fur* strain consistently exhibited lower bacterial density and reduced EPS signal in the stages associated with matrix maturation, i.e., 24 and 48 h (Figure 4 and Figure 5). The Δ*fur* biofilms displayed lower total WGA-associated fluorescence at later time points. Because WGA recognizes GlcNAc-containing material as well as another polymerized monosaccharide, and because the mutant simultaneously exhibited lower bacterial biomass, these observations are most conservatively interpreted as reduced accumulation of EPS-associated polysaccharides at the biofilm level. Whether Fur specifically affects PNAG production on a per-cell basis remains to be established. The absence of consolidated microcolonies and reduced EPS is consistent with a scenario in which Fur regulates the production of components essential for matrix formation, either directly or through downstream effectors. In this context, in bacteria such as *Y. pestis*, Fur regulates EPS production indirectly by modulating genes involved in c-di-GMP metabolism, such as diguanylate cyclases (DGCs) and phosphodiesterases (PDEs) [14]. Conversely, the absence of Fur in *Xanthomonas vesicatoria* impairs exopolysaccharide production [31], a phenotype shared with the *Y. ruckeri* ∆*fur* strain, but opposite to that of *Y. pestis*.

Regarding the observed reduced levels of EPS-associated GlcNAc (or another) at 48 h in the wild-type biofilm, apparently similar to those of the ∆*fur* strain (Figure 5), we previously reported that the fluorescence emission of Alexa Fluor dye can be attenuated by amino acids present in the extracellular environment of mature biofilms, thus affecting signal detection [7]. These phenomena may partly explain the apparent decrease in EPS-associated GlcNAc observed at 48 h in the wild-type strain. However, the apparent decrease in WGA-associated fluorescence at 48 h should be interpreted cautiously, since changes in signal intensity may also reflect alterations in matrix abundance, composition, spatial accessibility, or staining efficiency during late biofilm development.

The synthesis and export of PNAG into the extracellular matrix depend on the tight regulation of the *hms* gene family in the *Yersinia* genus, particularly the *hmsHFRS* operon, which encodes the machinery responsible for PNAG biosynthesis and transport [32]. The expression levels of these genes are closely linked to the intracellular concentration of the second messenger c-di-GMP. Elevated levels of c-di-GMP promote the synthesis and export of PNAG and biofilm development, whereas lower levels favor the transition to a planktonic lifestyle [30]. Based on the decreased EPS-associated GlcNAc levels observed in the Δ*fur* strain, reduced c-di-GMP levels were anticipated for the mutant strain in comparison with the wild-type. However, we found no detectable genotype-dependent difference in bulk intracellular c-di-GMP levels (Figure 6) or in the analyzed *hms* transcripts (Figure 9). Thus, the impaired Δ*fur* biofilm phenotype does not appear to be accompanied by a major alteration in the global c-di-GMP pool. This finding contrasts with observations in other species, such as *V. cholerae*, where Fur regulates the expression of *hmsP*, encoding a phosphodiesterase [15]. In *Y. pestis*, Fur directly represses *hmsT* transcription, encoding a diguanylate cyclase, and thereby decreases the intracellular c-di-GMP pool, whereas no transcriptional effect on *hmsHFRS* was detected [14]. These observations argue against a major Fur-dependent change in the bulk c-di-GMP pool under the conditions tested. However, growing evidence demonstrates that c-di-GMP signaling operates through highly compartmentalized, localized pools controlled by specific diguanylate cyclases and phosphodiesterases [33,34,35]. In *E. coli*, deletion of individual DGCs or PDEs produced significant biofilm phenotypes without altering total cellular c-di-GMP, and localized DGC activity adjacent to effector proteins has been shown to be both necessary and sufficient for exopolysaccharide biosynthesis without measurable changes in the global c-di-GMP pool [34]. Moreover, real-time spatial mapping has revealed non-uniform c-di-GMP distribution within biofilms, with concentrated hot spots at colony boundaries [36]. Therefore, the absence of detectable bulk differences observed here does not exclude the possibility that Fur deletion alters localized c-di-GMP signaling modules relevant to biofilm architecture in *Y. ruckeri*. Determining whether specific DGCs or PDEs are affected by *fur* deletion, and whether localized c-di-GMP pools are altered, represents an important objective for future studies, potentially employing c-di-GMP biosensors or activity assays for individual signaling components [37].

### 3.3. Transcriptomic Analysis of the ∆fur Strain: Regulatory Implications in the Biofilm Cycle

**Figure 11:**
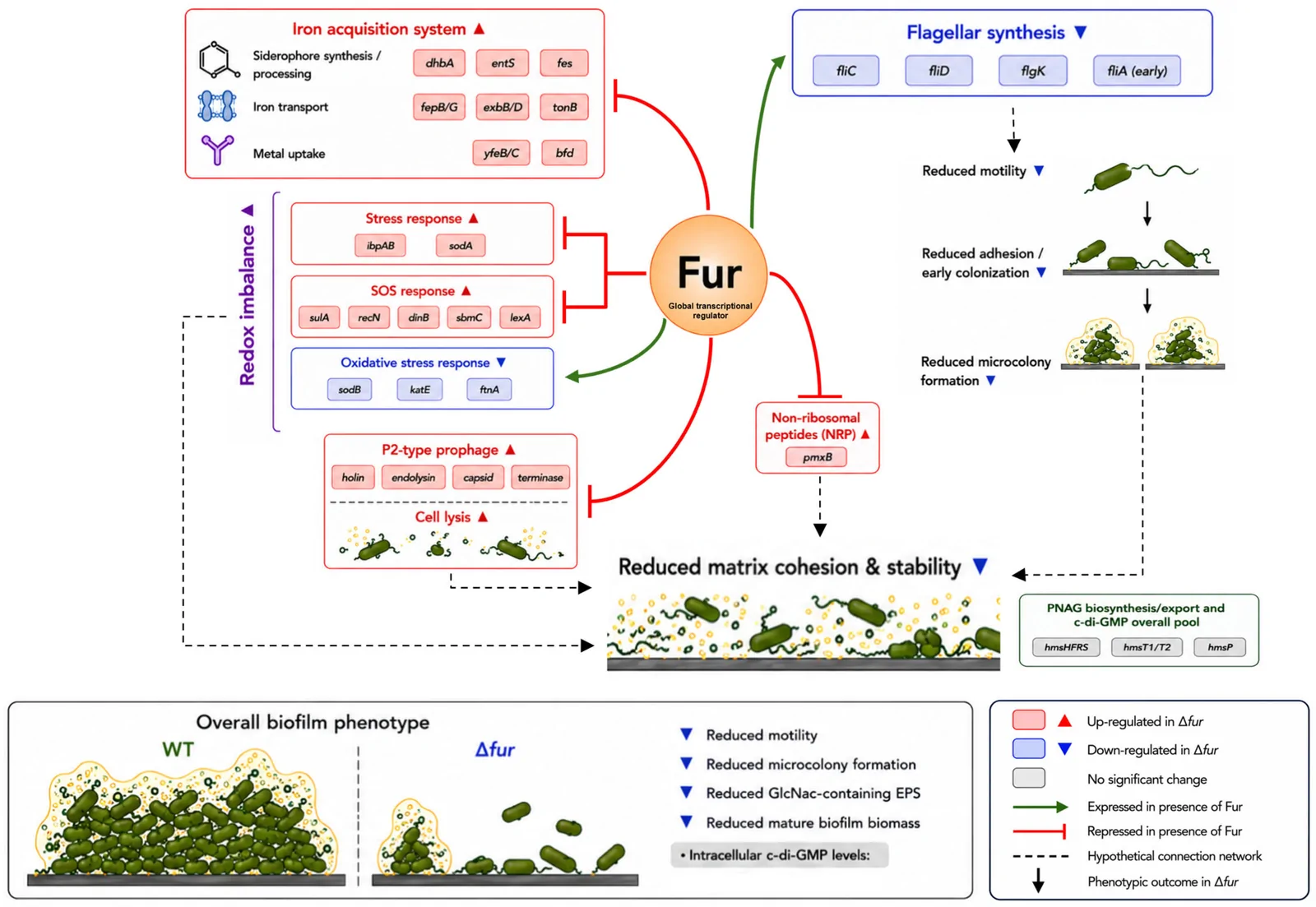
Working model of Fur-dependent phenotypic and transcriptional changes associated with *Y. ruckeri* biofilm development. This figure was generated with the assistance of ChatGPT, powered by GPT-5.6 Sol (OpenAI, San Francisco, CA, USA).

The transcriptomic analyses revealed extensive Fur-dependent changes in gene expression during biofilm development. These differences likely include a combination of direct and indirect effects of *fur* deletion and, particularly at later time points, transcriptional consequences of the distinct physiological and structural states reached by the wild-type and Δ*fur* populations. These observations are summarized in Figure 11 as a working model integrating Fur-dependent phenotypes, transcriptional associations, and testable mechanistic hypotheses. The working model does not imply direct Fur binding to the indicated genes unless such regulation has been independently demonstrated.

The transcriptomic profile reflects a disruption of iron homeostasis, with strong up-regulation of genes involved in siderophore biosynthesis and transport (*dhbA*, *entS*, *fepB*, *fepG*, *fes*, and the isochorismate synthase feeding catecholate synthesis), TonB-dependent energization (*exbB*, *exbD*, *tonB*), and the iron/manganese ABC transporter (*yfeB*, *yfeC*). In the absence of the iron-responsive repressor, this uncontrolled activation of iron uptake is expected to promote intracellular iron accumulation and, consequently, oxidative stress [25,38,39]. Consistent with this, the Δ*fur* strain displayed a characteristic remodeling of its oxidative-stress and iron-storage machinery: the iron-dependent superoxide dismutase *sodB*, the ferritin *ftnA*, and the catalase *katE* were down-regulated, whereas the manganese-dependent superoxide dismutase *sodA* and the chaperones of proteotoxic stress response *ibpAB* were up-regulated (Figure 8 and Figure 9). This *sodB*-to-*sodA* switch, together with the repression of iron-storage and catalase functions, is a hallmark of iron-homeostasis imbalance and points to an incomplete or unbalanced antioxidant response, which may leave the Δ*fur* strain more vulnerable to the oxidative burden generated under iron imbalance. This vulnerability is consistent with the reduced expression of oxidative-response functions detected in the GO enrichment analysis (Figure 7) and may contribute, at least in part, to the impaired biofilm-forming capacity of the mutant strain. The Δ*fur* strain also displayed a significant activation of the SOS response, with up-regulation of *sulA*, *recN*, *dinB*, *sbmC*, and *lexA*, which peaked at 12 and 48 h (Figure 8). Since the SOS response is triggered by oxidative damage to DNA, it is also plausible to suggest a coherent cascade in which iron-driven oxidative stress promotes DNA damage and SOS activation. However, as the iron and redox status of the biofilms was not directly measured, the increased expression of iron-acquisition and stress response systems in the Δ*fur* strain is most parsimoniously interpreted as a result of loss of Fur-mediated repression rather than as evidence that the experimental biofilm environment itself was iron-altered.

On the other hand, iron availability can influence biofilm architecture through mechanisms that extend beyond siderophore-mediated acquisition. In *P. aeruginosa*, iron limitation alters the timing of rhamnolipid production and increases surface motility, resulting in flatter and less structured biofilms [40]. Whether the increased expression of siderophore-associated genes observed in the *Y. ruckeri* Δ*fur* strain contributes functionally to rhamnolipid biosynthesis and its subsequent biofilm phenotype remains to be determined.

The ∆*fur* strain also exhibited altered but differential expression profiles of genes for biosynthesis of methionine and aromatic amino acids, i.e., *metE* and *aroF*, respectively (Figure 8). Methionine, together with other amino acids such as glutamate and cysteine, has been reported to be required for biofilm maintenance and maturation, likely through its contribution to redox balance, protein synthesis, and matrix-associated processes [41,42]. In this context, it could be hypothesized that the altered profiles of *metE* and *metC* observed in the ∆*fur* strain may contribute to biofilm instability by limiting the availability of key metabolic inputs necessary for sustained matrix production. Conversely, aromatic amino acids, particularly D-tyrosine and D-tryptophan, have been described as biofilm dispersal signals in bacteria such as *P. aeruginosa* and *Staphylococcus aureus* [43]. Thus, the up-regulation of *aroF* in the ∆*fur* strain, a gene associated with the biosynthesis of aromatic amino acids, could also contribute to the phenotype of Δ*fur* strain, which is unable to form a mature biofilm. On the other hand, the transcriptomic analysis also revealed a strong and sustained up-regulation in the case of the non-ribosomal peptide gene *pmxB*. It is known that these types of molecules act as biosurfactants that lower surface tension and disrupt cell–matrix interactions, thereby triggering active biofilm dispersal and swarming motility [44,45]. In this context, it is also plausible to hypothesize that the endogenous overproduction of this non-ribosomal peptide occurs prematurely in the Δ*fur* strain, leading to a condition that sustainedly promotes a dispersed phenotype. Further studies are required to determine the chemical identity and biological activity of the corresponding metabolites, to then confirm their possible role in biofilm stability or dispersal in *Y. ruckeri*.

A prominent feature of the Δ*fur* transcriptome was the strong and coordinated induction of a specific resident prophage. The *Y. ruckeri* CD2 genome harbors several prophage regions, but only one, a contiguous P2-type element, was markedly up-regulated in the mutant, with roughly half of its genes (19 of 34) significantly induced at the early and intermediate stages (6 and 12 h; mean log2 fold-change ≈ 1.6), whereas the remaining prophage regions showed no coordinated response. The induced element included structural genes encoding the major capsid, terminase, tail sheath, tail tube, and baseplate proteins (individual log2 fold-changes between +2 and +4), together with its lytic module comprising an HP1-family holin and an endolysin (lysozyme). Prophage-mediated lysis is increasingly recognized as a fundamental contributor to biofilm development in diverse bacterial species [46,47]. For instance, prophage-mediated modulation of biofilm has been best characterized in *Shewanella oneidensis* MR-1, where two resident prophages, a lambdoid phage and Mu-like phage, modulate biofilm dynamics in this microorganism. The lambdoid prophage induction is triggered by elevated iron concentrations, leading to partial lytic induction within the biofilm community [48]. For its part, the induction of the Mu-like prophage enhances biofilm extracellular DNA (eDNA) and promotes biofilm formation [49]. Otherwise, in *E. faecalis*, induction of prophage 5 by the quorum sensing signal AI-2 or by ciprofloxacin causes release of phage particles and marked reduction in biofilm biomass [50]. In the case of *P. aeruginosa* PAO1, the filamentous prophage Pf4 undergoes conversion to infective form within biofilm microcolonies, triggering localized cell death and facilitating the dispersal of viable subpopulations [51].

The temporal profile of prophage gene induction observed in this study, peaking at 6 and 12 h but declining by 24 h (Figure 8 and Figure 9), is consistent with early-phase lysis contributing eDNA for matrix consolidation rather than late-stage dispersal. To further validate prophage functional activity in *Y. ruckeri*, transmission electron microscopy (TEM) evaluations were utilized, detecting mature phage-like structures in Δ*fur* supernatants (Figure S5). This evidence strongly argues in favor of explosive cell lysis mechanisms within the biofilm. Furthermore, we conducted phenotypic plaque assays utilizing Δ*fur* supernatant on wild-type lawns, revealing translucent halos that faded progressively upon serial dilution without resolving into discrete plaques (Figure S5). This specific fading-halo phenotype also argues in favor of the massive release of soluble matrix-degrading enzymes, such as depolymerases or endolysins, characteristic of population-level explosive lysis, since active viral replication is blocked by resident superinfection immunity [52,53,54,55]. Thus, we hypothesize that this evidence represents localized induction and stochastic, SOS-dependent suicidal events undertaken by bacterial subpopulations. In this context, it has been reported in other bacteria that explosive lysis events act as highly coordinated regulatory mechanisms, driving the massive release of extracellular DNA (eDNA) and cytosolic components directly into the surrounding matrix [52,53,54,55]. Future studies should test whether this resident prophage operates as a fundamental driver of biofilm structural modulation and/or biofilm dispersion in *Y. ruckeri*. To this end, it will be essential to generate a prophage-cured strain of *Y. ruckeri* to evaluate the formation of true lytic plaques upon susceptible bacterial lawns. In addition, future studies must delineate the interconnected regulatory hierarchy governing this viral induction, including potential involvement of SOS response pathways. Likewise, it will be necessary to clarify whether Fur modulates prophage activation directly or through intermediate effectors, and how this dynamic ultimately governs the bacterial biofilm cycle in this pathogen.

Regarding the limitations of this study, it is important to note that an important consideration when interpreting the transcriptomic data is that the wild-type and Δ*fur* populations were sampled at matched chronological time points but did not necessarily occupy equivalent physiological or developmental states. This distinction is particularly relevant at 48 h, when their biofilm architecture differs markedly. Consequently, some transcriptional differences may represent secondary consequences of altered biomass, nutrient availability, oxygen exposure, or biofilm organization rather than primary Fur-dependent regulatory events. On the other hand, the transcriptomic analyses identify Fur-dependent expression changes but do not distinguish direct Fur targets from indirect regulatory or physiological effects. Determining which targets are directly regulated by Fur through DNA-binding assays such as EMSA or ChIP-seq represents an important objective for future mechanistic studies. Additionally, the RNA-seq analysis was performed at 6, 12, and 48 h, capturing early, intermediate, and late biofilm phases; the 24 h time point, which represents a critical transition toward mature biofilm, was not included and constitutes a temporal gap that may have captured additional relevant transcriptional changes. Future time-resolved transcriptomic studies, including the 24 h time point, would complement and strengthen the present findings. Furthermore, the c-di-GMP quantification reported here relied on bulk intracellular measurements, which may not capture functionally relevant localized changes in compartmentalized c-di-GMP signaling pools. Finally, CV staining was normalized to total cellular protein, which is an improvement over CFU normalization but does not fully account for extracellular matrix material. Future studies may employ dry weight normalization.

## 4. Materials and Methods

### 4.1. Bacterial Strain and Culture Conditions

*E. coli* strains were routinely propagated in Luria–Bertani (LB) medium (US BIO, Salem, MA, USA) supplemented, when appropriate, with ampicillin (100 μg/mL; Winkler, Santiago, Chile) or kanamycin (50 μg/mL; Sigma-Aldrich, St. Louis, MO, USA). Cultures were incubated at 37 °C with continuous shaking until reaching an optical density at 600 nm (OD_600_) of 1.1. Solid LB medium was prepared by adding 2% (*w*/*v*) agar-agar (Winkler, Santiago, Chile) and the corresponding antibiotics.

*Y. ruckeri* strains were cultured in Tryptic Soy Broth (TSB; BD Difco™, New Jersey, MD, USA), consisting of 17 g/L pancreatic digest of casein, 3 g/L pancreatic digest of soybean meal, 5 g/L NaCl, 2.5 g/L K_2_HPO_4_, and 2.5 g/L dextrose. Cultures were grown at 26 °C under constant agitation to an OD_600_ of 1.1. Tryptic Soy Agar (TSA) plates were prepared by supplementing TSB with agar-agar (Winkler, Santiago, Chile) at final concentrations of 1.2%, 0.5%, or 0.3% (*w*/*v*), according to the requirements of each assay. For biofilm experiments, bacterial suspensions were standardized to an OD_600_ of 0.5 (approximately 4 × 10^8^ CFU/mL) before inoculation and incubated under static conditions at 22 °C.

### 4.2. Construction of the Δfur Mutant and Cis-Complemented Strain

An unmarked Δ*fur* mutant was generated by allelic exchange as previously described [11]. Briefly, approximately 500 bp DNA fragments upstream and downstream of the *fur* coding sequence were PCR-amplified from *Y. ruckeri* genomic DNA using primer pairs annealing outside the target *locus*. The two homologous regions were fused by overlap-extension PCR through a 10 bp overlapping sequence, yielding an approximately 1 kb deletion construct. Following digestion with EcoRI and SacI (Thermo Fisher Scientific, Waltham, MA, USA), the PCR product was cloned into the suicide vector pFOK [56] (pFOK was a gift from Dirk Bumann [Addgene plasmid #166651; http://n2t.net/addgene:166651, accessed on 4 July 2026; RRID:Addgene_166651]) using T4 DNA Ligase (Promega, Madison, WI, USA). Both the digested insert and vector were purified using the Gel Extraction Kit (Omega BioTek, Norcross, GA, USA) before ligation.

The recombinant plasmid was introduced into *E. coli* S17-λpir and mobilized into *Y. ruckeri* by conjugation as previously described [11]. Double-crossover recombinants were selected by counterselection on TSA plates supplemented with 10% (*w*/*v*) sucrose (Merck, Darmstadt, Germany) and anhydrotetracycline (10 μg/mL; AppliChem, Darmstadt, Germany). Candidate Δ*fur* mutants were identified by loss of kanamycin resistance and verified by PCR using oligos flanking the *fur* locus (Supplementary Table S1).

For *cis*-complementation, an approximately 1 kb DNA fragment containing the complete wild-type *fur* gene was amplified from *Y. ruckeri* genomic DNA and cloned into pFOK following the same cloning strategy described above. The resulting construct was transferred into the Δ*fur* mutant by conjugation, and allelic exchange was performed using the same counterselection procedure. Candidate complemented clones were identified by restoration of kanamycin sensitivity and confirmed by PCR using oligos annealing outside the *fur* locus (Supplementary Table S1).

### 4.3. Motility Assays

Swimming and swarming motility were evaluated on TSA plates containing 0.3% and 0.5% (*w*/*v*) agar, respectively. *Y. ruckeri* cultures were grown in TSB to an OD_600_ of 1.1, and 1 μL of each culture was inoculated onto the center of the corresponding agar plates. Plates were incubated at 26 °C for 24 h, after which motility halos were documented using a G:BOX Chemi XRQ imaging system (Syngene, Cambridge, UK).

### 4.4. Visualization of Bacterial Flagella by Transmission Electron Microscopy

*Y. ruckeri* strains were grown in TSB at 26 °C with shaking until reaching an OD_600_ of 0.5. Aliquots of 2 mL were collected and centrifuged at 4000 rpm for 5 min. The supernatant was discarded, and the bacterial pellet was gently resuspended in 1 mL of 2.5% (*v*/*v*) glutaraldehyde (Electron Microscopy Sciences, Hatfield, PA, USA) by gentle pipetting. Fixed samples were stored at 4 °C until further processing.

Sample preparation and transmission electron microscopy (TEM) analyses were performed at the Advanced Microscopy Unit (UMA), Pontificia Universidad Católica de Chile. Bacterial suspensions were deposited onto glow discharge-treated Formvar/carbon-coated copper grids and negatively stained with 2% (*w*/*v*) uranyl acetate prior to imaging. Specimens were examined using a TECNAI Transmission Electron Microscope (FEI Company, Hillsboro, OR, USA).

### 4.5. Biofilm Formation Assay

*Y. ruckeri* strains were cultured in TSB medium at 26 °C with shaking until reaching an OD_600_ of 1.1. Cultures were subsequently diluted to an OD_600_ of 0.5 in fresh TSB, and 0.5 mL aliquots were dispensed into 24-well plates containing sterile 12 mm silicate coverslips (Marienfeld, Lauda-Königshofen, Germany). Biofilms were allowed to develop under static conditions at 22 °C for 4, 6, 12, 24 and 48 h. At the end of the incubation period, coverslips were gently removed, washed twice by immersion in sterile phosphate-buffered saline (PBS) to eliminate non-adherent cells, and processed according to the requirements of each subsequent analysis.

### 4.6. Crystal Violet Staining Assay

Biofilms generated as described in Section 4.5 were quantified by crystal violet staining. Silicate coverslips were fixed with 200 μL of 100% (*v*/*v*) methanol (Winkler, Santiago, Chile) for 10 min and then transferred to a new 24-well plate containing 1 mL of 1% (*v*/*v*) Crystal Violet solution (Winkler, Santiago, Chile). After staining for 20 min at room temperature, the silicate coverslips were washed twice by gentle immersion in sterile phosphate-buffered saline (PBS), air-dried, and the retained dye was solubilized with 500 μL of 33% (*v*/*v*) acetic acid (Winkler, Santiago, Chile). The absorbance of the resulting solution was measured at 570 nm using a Synergy H1 Microplate Reader (BioTek Instruments, Winooski, VT, USA). For data analysis and graphical representation, absorbance values were normalized to total protein content, quantified by the Bradford assay from adhered cells recovered by applying three pulses of low-frequency sonication (Elma, Singen, Germany), for 30 s each one.

### 4.7. Biofilm Architecture Analysis by Scanning Electron Microscopy

Biofilms generated as described in Section 4.5 were processed for scanning electron microscopy (SEM). Coverslips were fixed with 500 μL of 2.5% (*v*/*v*) glutaraldehyde (Electron Microscopy Sciences, Hatfield, PA, USA) and transported to the Advanced Microscopy Unit (UMA), Pontificia Universidad Católica de Chile, where samples were subjected to critical point drying, gold sputter coating, and SEM imaging. Biofilm architecture was examined using a Hitachi TM3000 scanning electron microscope (Hitachi High-Tech, Tokyo, Japan).

### 4.8. Biofilm Analysis by Confocal Laser Scanning Microscopy

For confocal microscopy experiments, *Y. ruckeri* strains were transformed with the pDiGc plasmid [57] (pDiGc was a gift from Sophie Helaine & David Holden [Addgene plasmid #59322; http://n2t.net/addgene:59322, accessed on 4 July 2026; RRID:Addgene_59322]).

Biofilms generated as described in Section 4.5 were fixed with 4% (*v*/*v*) paraformaldehyde (Electron Microscopy Sciences, Hatfield, PA, USA) for 20 min at room temperature. Coverslips were then mounted on glass slides using 10 μL of Fluoromount-G mounting medium (Thermo Fisher, Waltham, MA, USA), air-dried for 10 min in the dark, and stored at 4 °C until imaging.

For visualization of extracellular polysaccharides, paraformaldehyde-fixed biofilms were stained with 100 μL of wheat germ agglutinin (WGA) conjugated to Alexa Fluor 647 (Thermo Fisher, Waltham, MA, USA) for 20 min at room temperature in the dark. Following two washes with sterile phosphate-buffered saline (PBS), coverslips were mounted on glass slides with 10 μL of Fluoromount-G mounting medium. Samples were examined using a Leica TCS SP8 confocal laser scanning microscope (Leica Microsystems GmbH, Wetzlar, Germany).

### 4.9. Quantification of Cyclic Diguanosine Monophosphate (c-di-GMP)

Intracellular c-di-GMP levels were quantified using a Cyclic-di-GMP Assay Kit (Lucerna Technologies, Brooklyn, NY, USA) according to the manufacturer’s instructions. Biofilms generated as described in Section 4.5 were washed twice with sterile phosphate-buffered saline (PBS), transferred to 24-well plates containing 500 μL of sterile PBS, and the attached cells were recovered by applying three 30 s pulses of low-frequency sonication (Elma, Singen, Germany). Aliquots (70 μL) of the resulting cell suspensions were processed together with serial dilutions of the c-di-GMP standard following the manufacturer’s protocol. After incubation for 30 min at room temperature in the dark, fluorescence was measured using a Synergy H1 Microplate Reader (BioTek Instruments, Winooski, VT, USA) with excitation and emission wavelengths of 469 and 501 nm, respectively. Intracellular c-di-GMP concentrations were determined from a standard calibration curve and normalized to the number of colony-forming units (CFU) recovered from each biofilm sample.

### 4.10. RNA-Seq Data Analysis

Biofilms generated as described in Section 4.5 were sampled after 6, 12, and 48 h of incubation. For each time point, biofilms from the WT and Δ*fur* strains were collected in biological triplicate, resulting in a total of 18 samples for transcriptomic analysis. Total RNA was extracted using the GeneJET™ RNA Purification Kit (Thermo Scientific™, Waltham, MA, USA) according to the manufacturer’s instructions. Residual genomic DNA was removed by treatment with DNase I (Thermo Scientific™, Waltham, MA, USA) for 90 min at 37 °C in a T960 thermal cycler (Heal Force, Shanghai, China). Complete DNA removal was verified by PCR amplification using oligos targeting the housekeeping gene *gyrA*. RNA libraries were prepared using the Illumina TruSeq Small RNA Kit and sequenced on an Illumina MiSeq platform (151 bp, single-end). Raw data are available in the Sequence Read Archive (SRA) under Bioproject PRJNA1162115. Raw reads were quality-trimmed with fastp (v1.3.6) and aligned to the *Y. ruckeri* strain CD2 genome (GCF_050360435.1) with Bowtie2 (v2.5.5). The genome was annotated with Bakta (v6.0), and reads were assigned to coding sequences with featureCounts (Subread v2.1.0), yielding a CDS-level count matrix for 18 libraries (two genotypes × three biofilm stages × three biological replicates). Genes with fewer than 10 counts in at least three samples were removed.

Differential expression was analyzed in R (v4.5.2) with DESeq2 (v1.50.2), combining genotype and biofilm stage into a single grouping factor and extracting pairwise contrasts with the Wald test. Log2 fold changes were shrunk with the ashr method, and genes with an adjusted *p*-value ≤ 0.05 and |log2 fold-change| ≥ 1 were considered differentially expressed. Functional over-representation analysis of GO terms, COG categories, and KEGG pathways was performed with clusterProfiler (v4.18.4), using annotations derived from Bakta and KEGG. To separate stable from stage-dependent Fur effects, a likelihood-ratio test contrasting the full model (~genotype + stage + genotype:stage) against the reduced model (~genotype + stage) was applied. The sequencing data are available under BioProject accession PRJNA1162115, encompassing SRA run accessions SRR40041184 through SRR40041201.

### 4.11. RT-qPCR Analysis

Biofilms generated as described in Section 4.5 were collected after 4, 6, 12, 24, and 48 h of incubation for gene expression analysis. Total RNA was extracted using TRIzol reagent (Invitrogen™, Waltham, MA, USA) according to the manufacturer’s instructions. Residual genomic DNA was removed by treatment with DNase I (Thermo Scientific™, Waltham, MA, USA) for 90 min at 37 °C in a T960 thermal cycler (Heal Force, Shanghai, China). Complete DNA removal was confirmed by PCR amplification using oligos targeting the housekeeping gene *gyrA*. First-strand cDNA was synthesized from 1 μg of total RNA using gene-specific oligos (10 μM each) and M-MLV Reverse Transcriptase (Promega, Madison, WI, USA), following the manufacturer’s instructions. Quantitative PCR was performed using the KAPA SYBR^®^ FAST qPCR Kit (KAPA Biosystems, Wilmington, MA, USA) on an AriaMx Real-Time PCR System (Agilent Technologies, Santa Clara, CA, USA). Relative gene expression levels were normalized to the housekeeping gene *gyrA*.

### 4.12. Construction of Transcriptional Fusions

The native promoter regions from positions +1 to −500 of *fliA*, *pmxB*, *fes* and *sodB* genes were amplified by PCR with the oligos included in Table S1 using genomic DNA from *Y. ruckeri* CD2 strain. The restriction sites *XhoI* and *HindIII*, at the ends of the DNA fragments, were introduced by the PCR primers (underlined sequences in Table S1) and digested with the corresponding enzymes. The digested PCR product was cloned into the multiple cloning site (MCS) of the β-galactosidase reporter vector pLacZ-Basic (Clontech Laboratories, Inc., Mountain View, CA, USA; GenBank accession number U13184), generating the vectors P-lacZ. The constructs were transformed into wild-type and Δfur strains. To evaluate activity, cells were cultured under biofilm-formation conditions. β-galactosidase activity was determined as previously described [58].

### 4.13. Statistics

Statistical analyses were performed using GraphPad Prism v10 (GraphPad Software, San Diego, CA, USA) and R v4.3.2 (R Foundation for Statistical Computing). Data normality was assessed using the Shapiro–Wilk test and homogeneity of variances using Levene’s test. For normally distributed data with equal variances, parametric comparisons were conducted using Student’s *t*-test (two-group comparisons) or two-way ANOVA (factorial designs with two independent variables) as appropriate. When two-way ANOVA indicated significant main effects or interactions, Tukey’s Honestly Significant Difference (HSD) post hoc test was applied to perform pairwise multiple comparisons between group means while controlling the family-wise error rate. For non-normally distributed data or count data (e.g., number of flagella per cell), the nonparametric Mann–Whitney U test was applied. Differences were considered statistically significant when *p* < 0.05. Statistical significance is indicated in figures as: ns (*p* ≥ 0.05), * (*p* < 0.05), ** (*p* < 0.01), *** (*p* < 0.001), and **** (*p* < 0.0001).

## 5. Conclusions and Future Perspectives

The present study was designed to provide global, integrative characterization of phenotypic and transcriptomic consequences following *fur* deletion during *Y. ruckeri* biofilm development. Collectively, our findings identify Fur as an important contributor to the normal progression toward mature and spatially organized *Y. ruckeri* biofilms. Loss of *fur* is associated with reduced motility and flagellar abundance, impaired multicellular organization and matrix-associated accumulation, and extensive transcriptional reprogramming. These findings are particularly relevant in the context of marine pathogens, where biofilm formation constitutes a critical strategy for environmental persistence, treatment resistance, and colonization of surfaces associated with aquaculture systems. The Fur-dependent pathways and factors identified here, particularly those associated with flagellar functions, stress responses, and extracellular matrix stability, provide candidate processes for future mechanistic studies and may ultimately help identify strategies to interfere with the sessile persistence of *Y. ruckeri* in aquaculture environments.

## Data Availability

The sequencing data are available under BioProject accession PRJNA1162115, encompassing SRA run accessions SRR40041184 through SRR40041201.

## Supplementary Materials

The following supporting information can be downloaded at: https://www.mdpi.com/article/10.3390/ijms27188413/s1.
