# Supplementary material for: Participation of the Transcriptional Regulator Fur in Modulating Biofilm Formation of the Salmonid Pathogen Yersinia ruckeri: Beyond Iron Homeostasis
Source: Int J Mol Sci. 2026 Sep 21;27(18):8413. doi: 10.3390/ijms27188413 (PMC13607417; doi:10.3390/ijms27188413)

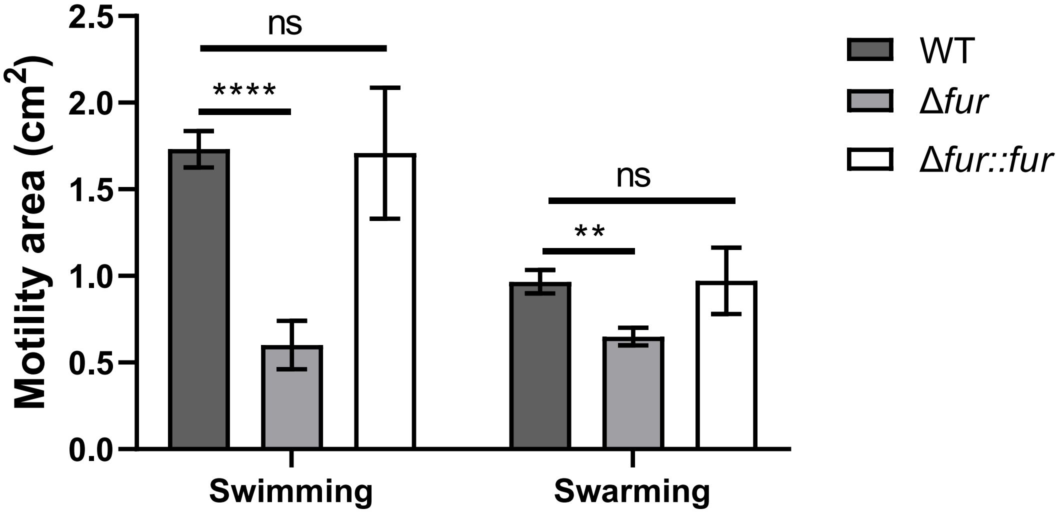

Figure S1: Assessment of motility in Δ*fur* complemented strain (Δ*fur*::*fur*). Swimming and swarming motility assays were performed by inoculating 1 μL of overnight cultures onto plates containing 0.3% (w/v) and 0.5% (w/v) agar, respectively. Bar graph showing motility areas calculated from migration zone diameters. Data is presented as mean ± standard error (*n* = 9). Statistical analysis was performed using two-way ANOVA (***p* < 0.05; *****p* < 0.0001).

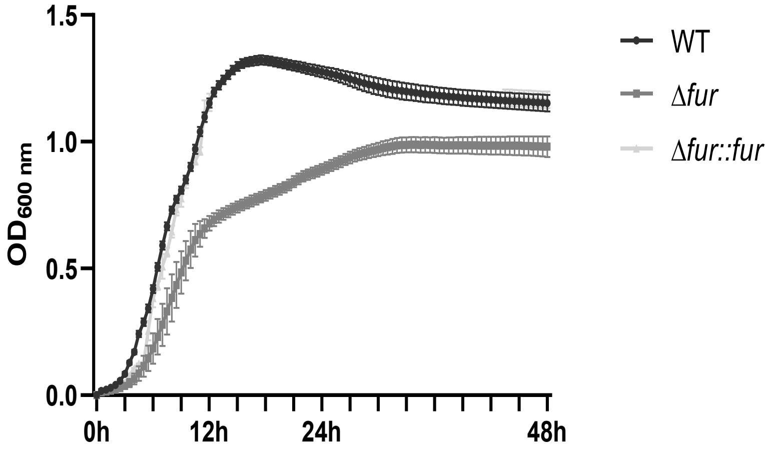

Figure S2: Growth Curves of WT and Δ*fur* strains. Curves were generated from overnight cultures adjusted to an initial optical density (OD_{600}) of 0.04 (~3.2 × 10^7 CFU/mL) following washing with sterile PBS. Cells were resuspended in 500 μL of TSB and

incubated in 24 well plates. OD_{600} measurements were taken every 30 minutes using a Synergy H1 plate reader (BioTek) under continuous shaking at 26 °C. Each curve represents the average of nine independent replicates (n = 9). Statistical analysis was performed using two-way ANOVA.

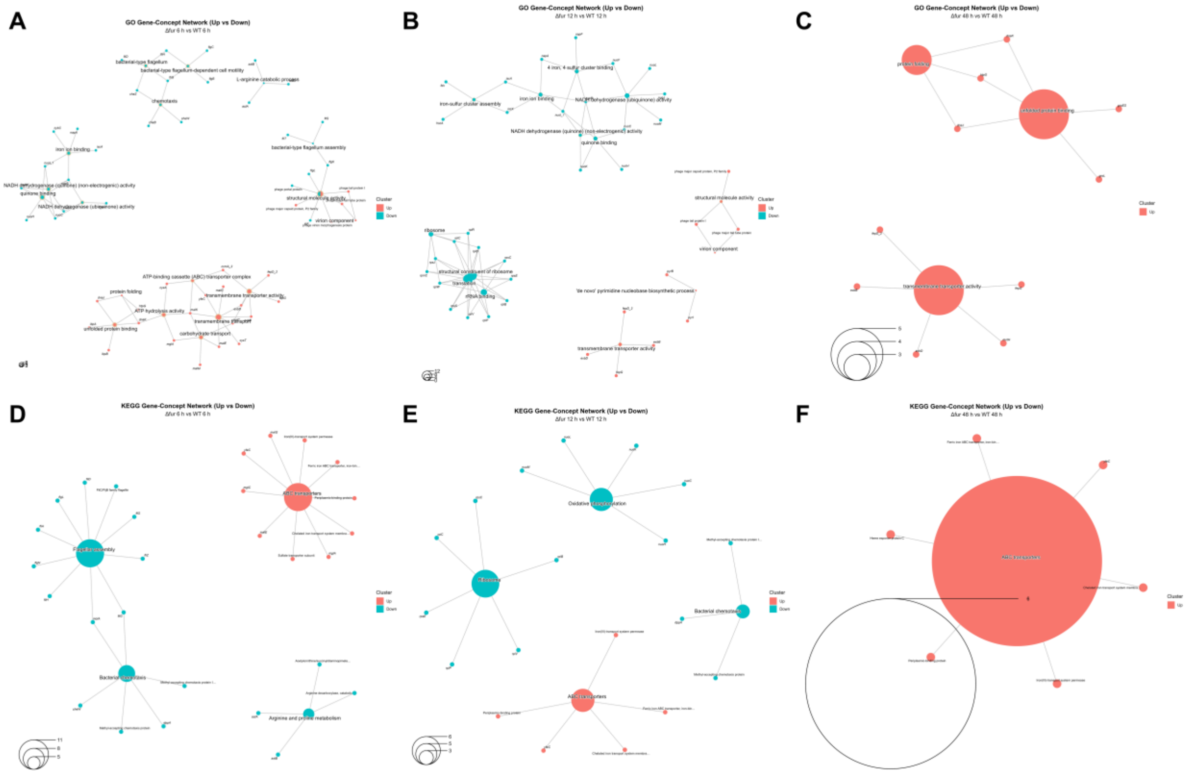

Figure S3: Gene-concept networks of enriched GO terms and KEGG pathways in the Δ*fur* strain during biofilm formation. Cnetplots showing the genes associated with enriched GO terms (A–C) and KEGG pathways (D–F) for the up- (red) and down-regulated (blue) gene sets of the Δ*fur* strain relative to the wild-type at 6 h (A, D), 12 h (B, E), and 48 h (C, F).

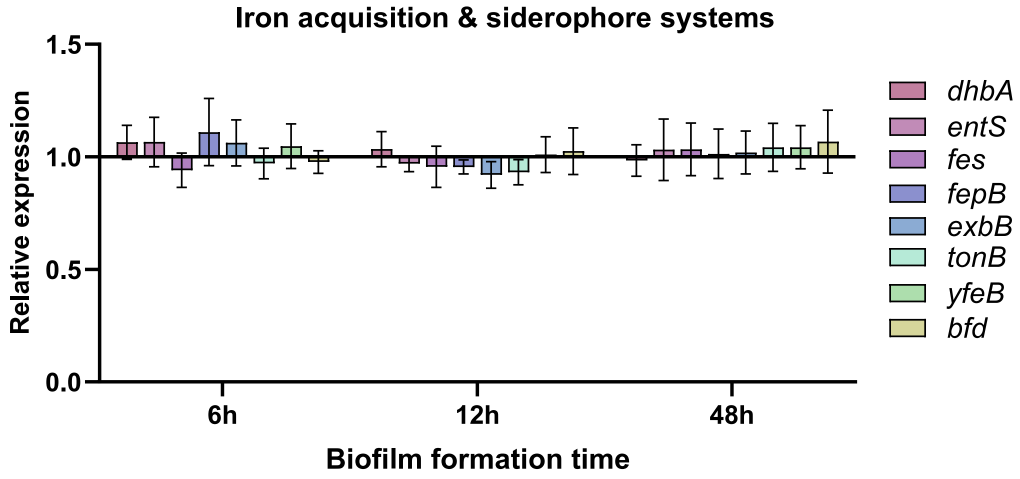

Iron acquisition & siderophore systems
1.5
1.0
0.5
0.0
Relative expression
6h
12h
48h
Biofilm formation time
dhbA
entS
fes
fepB
exbB
tonB
yfeB
bfd

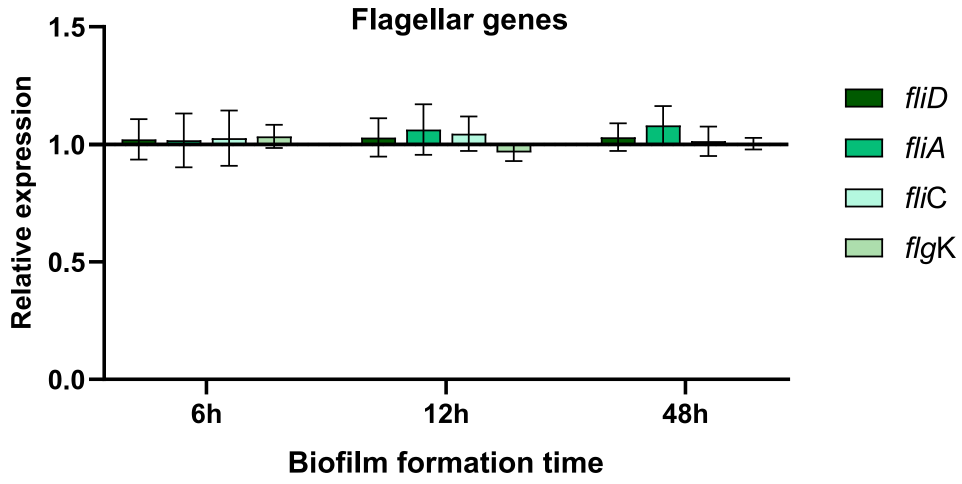

Flagellar genes
1.5
1.0
0.5
0.0
Relative expression
6h
12h
48h
Biofilm formation time
fliD
fliA
fliC
flgK

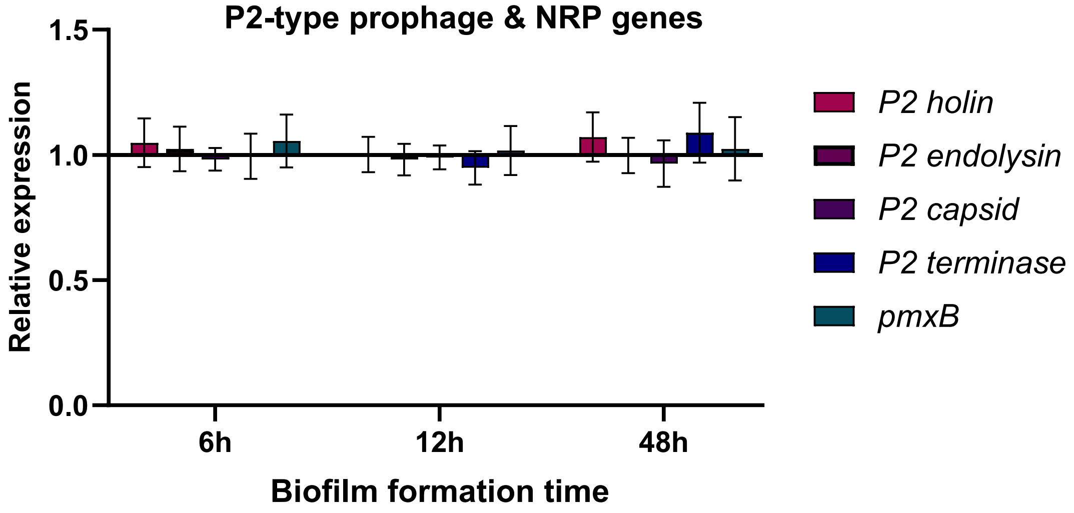

P2-type prophage & NRP genes
1.5
1.0
0.5
0.0
Relative expression
6h
12h
48h
Biofilm formation time
P2 holin
P2 endolysin
P2 capsid
P2 terminase
pmxB

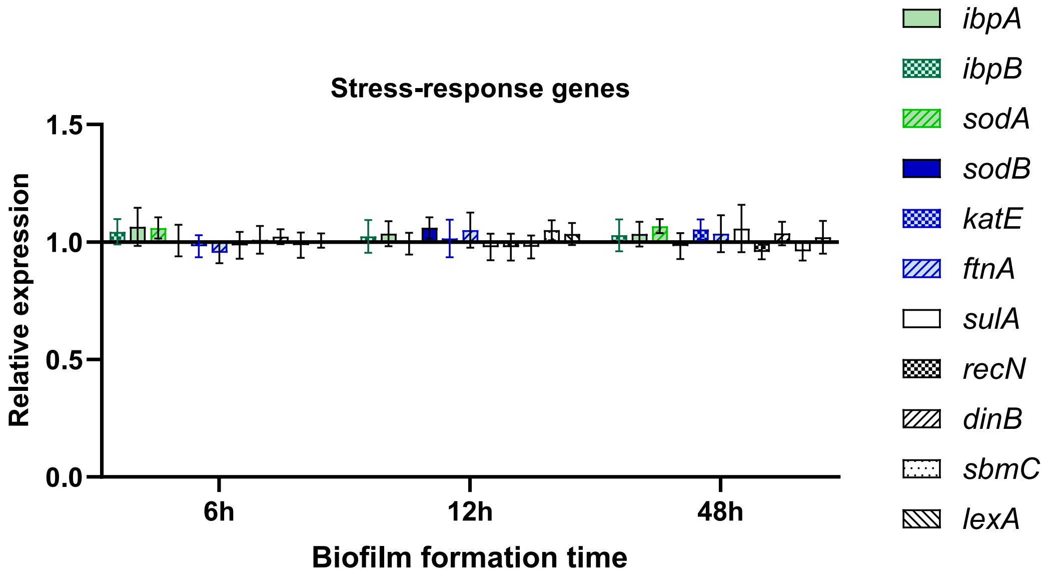

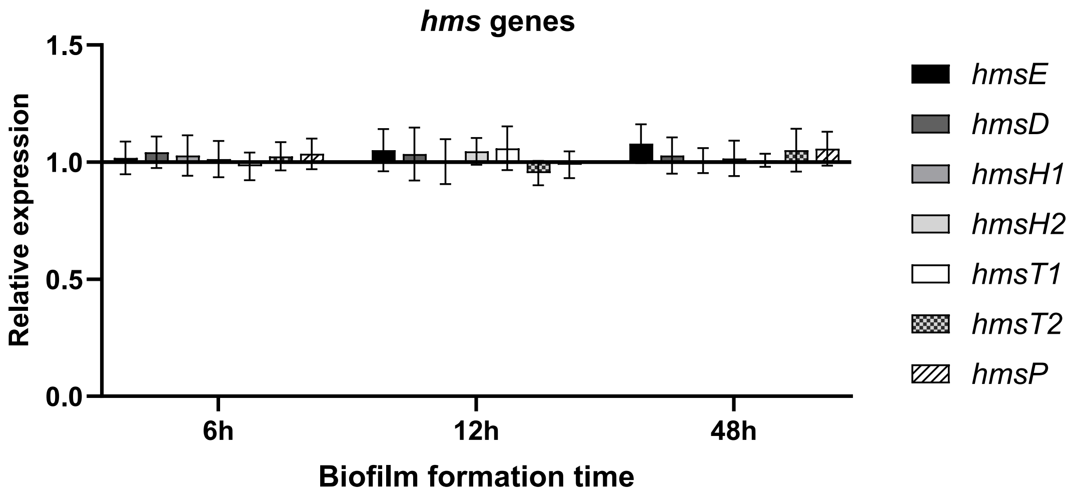

Figure S4: Relative gene expression analysis in the Δ*fur* complemented strain of *Y. ruckeri* during biofilm formation. Wild-type (WT) and Δ*fur* complemented strain were grown without shaking at 22 °C on covers for the indicated times. Adherent cells were recovered by low-frequency sonication and subjected to RNA extraction. Gene expression was quantified by RT-qPCR and is shown relative to WT.

A

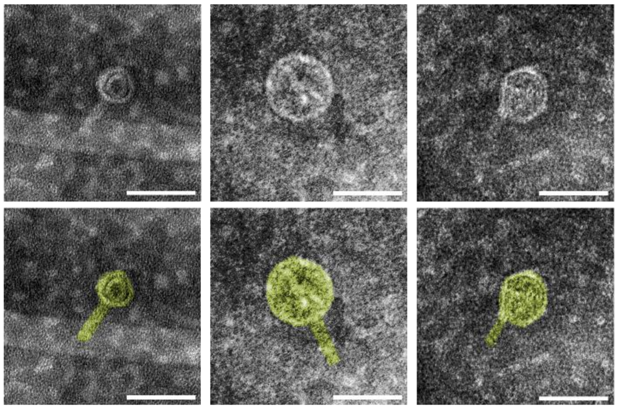

B

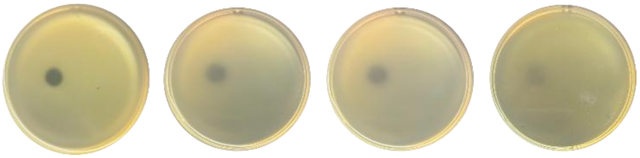

Figure S5: Transmission electron microscopy (TEM) and phenotypic plaque assays of Δ*fur* supernatants. The Δ*fur* strain was grown without shaking at 22 °C on covers for 12h and the supernatant was recovered to analyze it by TEM and for plaque assays. **(A)** Representative TEM micrographs showing four bacteriophage particles displaying a capsid-like head and an associated tail structure. The upper row shows the original grayscale micrographs, whereas the corresponding images in the lower row were digitally pseudocolored in green to facilitate visualization of the phage morphology. Pseudocoloring was performed for illustrative purposes only and does not represent an additional experimental signal or measurement. Scale bars, 50 nm. **(B)** Plaque assays on a lawn of the wild-type strain with three serial dilutions, from left to right. Additional uncropped images are shown below.

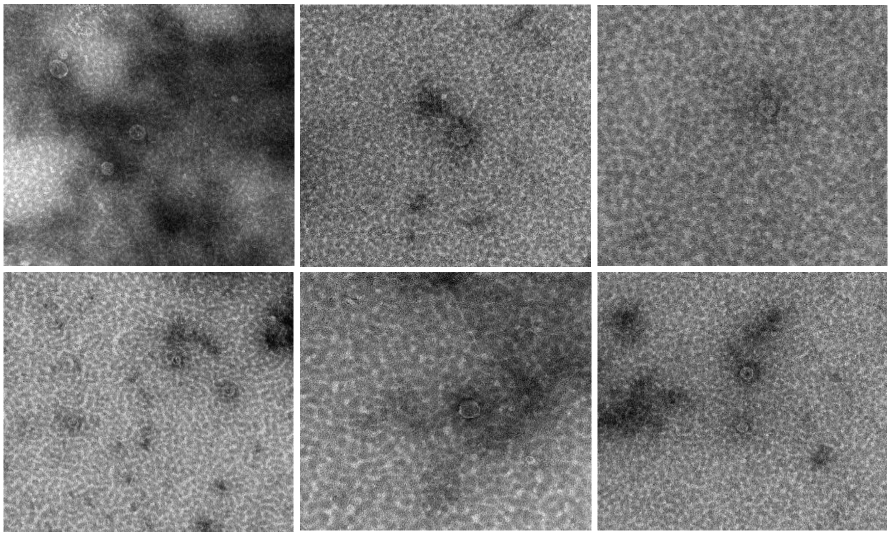

Supplement: Supplementary file 1 [file ijms-27-08413-s001.zip › Supplemmentary Figures Peñaloza et al 2026.pdf]
